# Quality, Readability, Understandability, and Actionability of AI Chatbots’ Responses to Dental Patient Questions: A Comparative Evaluation

**DOI:** 10.3390/diagnostics16203430

**Published:** 2026-10-21

**Authors:** Nebiha Gozde Ispir, Gokalp Aslan, Gulsun Akay

**Affiliations:** Department of Oral and Dentomaxillofacial Radiology, Gazi University, Ankara 06490, Turkey; gokalpaslan@gazi.edu.tr (G.A.); gulsunakay@gazi.edu.tr (G.A.)

**Keywords:** artificial intelligence, large language model, dental patient education, multidimensional evaluation, readability, PEMAT, effect size, Pareto analysis

## Abstract

**Background/Objectives**: Artificial intelligence (AI) chatbots are increasingly used to obtain oral health information, yet prior dental studies have already evaluated overlapping domains such as accuracy, quality, readability, understandability, and actionability. This study applied and extended a multidimensional evaluation approach to compare AI-generated answers to dental patient questions, with emphasis on Turkish-language readability, standard PEMAT-P applicability, effect-size and uncertainty reporting, and inter-rater reliability. **Methods**: Thirty-three AI-assisted, expert-curated questions were submitted to ChatGPT 5.2, Gemini, DeepSeek V3, and Grok 3, yielding 132 responses. Three dental evaluators independently assessed clinical accuracy, Global Quality Score (GQS), understandability (PEMAT-U), and actionability (PEMAT-A). PEMAT-P scores were calculated using the standard item structure and item-specific applicability rules, with items coded as not applicable (N/A) excluded from the denominator. Readability was calculated using a deterministic, rule-based implementation of the Ateşman formula. Model comparisons used the Friedman test, Bonferroni-adjusted Wilcoxon signed-rank tests, effect sizes, bootstrap confidence intervals, and two-way random-effects intraclass correlation coefficients. An exploratory Pareto analysis assessed multidimensional dominance without imposing arbitrary weights. **Results**: Significant model differences were observed for all five dimensions (all *p* ≤ 0.002). Gemini had the highest mean GQS, clinical accuracy, PEMAT-U, and PEMAT-A scores, but its Ateşman readability score was significantly lower than those of ChatGPT, DeepSeek, and Grok. No significant readability differences were observed among the latter three models. Average-measure reliability was poor for GQS and clinical accuracy and moderate for PEMAT-U and PEMAT-A. No model dominated another across all five dimensions. **Conclusions**: The chatbots demonstrated model-specific strengths rather than uniform superiority. Their performance should be interpreted multidimensionally, together with effect magnitude, uncertainty, and inter-rater reliability, rather than through a single overall ranking.

## 1. Introduction

Recent advances in artificial intelligence (AI) technologies have enabled the development of large language models (LLMs) with human-like natural language processing capabilities [1,2]. These models have emerged as alternative sources of health information by providing access to online knowledge [2]. AI chatbots such as ChatGPT (OpenAI), Gemini (Google), DeepSeek (DeepSeek AI), and Grok (xAI) are becoming increasingly popular because they can provide rapid and accessible responses to users’ health-related questions [3]. These models differ in terms of their training datasets, architectural designs, and ability to access real-time information.

With the widespread adoption of these chatbots, an increasing number of patients have begun directing medical and health-related questions to these platforms [3]. However, these systems are not specifically optimized for answering medical questions, and their training data may include not only peer-reviewed scientific literature but also medical misinformation, health advice generated by non-experts, biased content, and outdated clinical guidelines [4]. Consequently, concerns have arisen regarding the accuracy, reliability, readability, and actionability of the information they provide. On the other hand, AI chatbots are increasingly being integrated into various aspects of healthcare, including clinical decision support, patient education, medical documentation, and patient–physician communication [5,6]. Owing to their advanced natural language processing capabilities, these systems can deliver real-time, user-friendly responses to patients’ health-related inquiries [6]. Furthermore, by offering innovative solutions to a variety of clinical challenges, AI chatbots have the potential to enhance the efficiency of healthcare professionals in both medicine and dentistry [7].

Poor oral health remains a prevalent public health problem in many parts of the world [8]. In addition, the high cost of dental services may limit access to treatment and contribute to delays in obtaining necessary dental care [9]. As a result, patients are increasingly using AI chatbots to seek dental information and advice before, during, and after dental appointments or procedures [10]. Inaccurate, incomplete, or poorly readable AI-generated responses may delay treatment-seeking behavior, increase patient anxiety, or encourage inappropriate health-related decisions. This issue is particularly important in dentistry, where conditions such as pain, infection, and postoperative complications may require timely intervention. Therefore, a systematic evaluation of the responses generated by AI chatbots to dental patient questions is clinically warranted.

Previous dental studies have already used overlapping multidimensional approaches, so the present study does not claim that combining accuracy, quality, readability, understandability, and actionability constitutes a new instrument. Özcivelek and Özcan [11] evaluated accuracy, GQS, English-language readability, and PEMAT-P understandability/actionability for prosthodontic patient questions; Sivaramakrishnan et al. [12] evaluated readability, PEMAT understandability/actionability, and inter-rater agreement for four dental patient education scenarios; and Wu et al. [13] used a five-dimension expert evaluation with inter-rater reliability and multivariate analysis in implantology. The gap addressed here is therefore narrower: a broad Turkish-language dental patient question set spanning multiple clinical categories, deterministic Ateşman readability processing, standard applicability-based PEMAT-P scoring, matched-question effect-size and bootstrap uncertainty reporting, explicit ICC reporting for both single and averaged ratings, and an exploratory non-weighted analysis of cross-domain trade-offs. Table 1 summarizes the methodological overlap and distinctions among these studies.

Clinical accuracy, global quality, readability, understandability, and actionability represent related but non-equivalent properties of patient-oriented information. The Ateşman formula evaluates Turkish-language readability through word- and sentence-level characteristics [14], whereas the Patient Education Materials Assessment Tool distinguishes whether information can be understood from whether users can identify actions to take [15]. Likewise, statistically significant differences among models should be considered together with the reliability of the human ratings on which those comparisons are based [16]. Collapsing these dimensions into an arbitrarily weighted composite score could conceal clinically meaningful trade-offs and produce rankings that depend on unvalidated weighting decisions.

Accordingly, the present study aimed to compare the clinical accuracy, global quality, readability, understandability, and actionability of responses generated by ChatGPT 5.2, Gemini, DeepSeek V3, and Grok 3 to a standardized, AI-assisted, expert-curated set of Turkish-language dental patient questions covering a broad range of oral and dental health concerns. The contribution is not the creation of a new measurement instrument or a simple update of model versions; rather, it lies in combining Turkish-specific deterministic readability assessment, standard PEMAT-P applicability rules, reliability-aware interpretation, matched non-parametric effect sizes with bootstrap confidence intervals, and exploratory weight-free trade-off analysis under controlled query conditions. The study was designed to characterize the sampled outputs under these conditions rather than reproduce the full linguistic and contextual variability of authentic patient-generated questions.

## 2. Materials and Methods

### 2.1. Study Design

No animal experiments or personal/clinical data from human participants were used in this study. Therefore, the study was designed as a cross-sectional in silico investigation conducted in a digital environment and did not require ethics committee approval.

Four different AI chatbot models—ChatGPT 5.2 (OpenAI), Gemini (Google), DeepSeek V3 (DeepSeek AI), and Grok 3 (xAI)—were evaluated. To construct a standardized set of dental patient questions, each model was initially prompted with the question: ‘What questions do individuals most frequently ask you about oral and dental health?’ The resulting question pools were compiled and independently screened by two licensed dentists with clinical and academic experience in oral diagnosis, oral and maxillofacial radiology, and patient education: an oral and maxillofacial radiologist with more than 10 years of clinical experience and an oral and maxillofacial radiology resident with at least 5 years of clinical experience. The screening considered patient orientation, clinical relevance, clarity as a standalone question, duplication/semantic overlap, and whether a question appeared in more than one model-generated pool. Identical or substantially overlapping questions were merged, and questions generated by only one chatbot were excluded to reduce model-specific idiosyncrasy. After the independent screening, the two reviewers jointly finalized the wording and clinical categorization of the retained questions. This process yielded 33 questions. The set was intended as a standardized, clinically relevant sample for controlled comparison, not an exhaustive or epidemiologically validated representation of naturally occurring patient inquiries.

Real-world patient-generated questions were not used because the primary objective was to compare the models under identical query conditions without collecting or processing personal patient data. Questions drawn from consultations, records, forums, or social media may contain identifiable information, institution-specific context, misspellings, incomplete histories, ambiguous wording, emotional framing, and highly individualized clinical details. Their exclusion improved experimental standardization but reduced ecological validity. Because the test set was partly shaped by AI-generated question pools and then refined by experts, model-dependent selection bias and circularity cannot be excluded. The findings should therefore be interpreted as performance under standardized expert-curated conditions rather than as a direct estimate of performance across the diversity of authentic patient discourse.

To provide clinical context, the final 33 questions were descriptively categorized according to their primary topic.

The following categories were established:

1. Tooth Pain and Infections;
2. Periodontal and Gingival Problems;
3. Tooth Extraction and Post-Extraction Care;
4. Impacted Teeth and Third Molars;
5. Dental Implants, Bridges, and Prostheses;
6. Dental Esthetics and Tooth Whitening;
7. Pediatric Dental Problems;
8. General Information and Preventive Dentistry.

**Figure 1:**
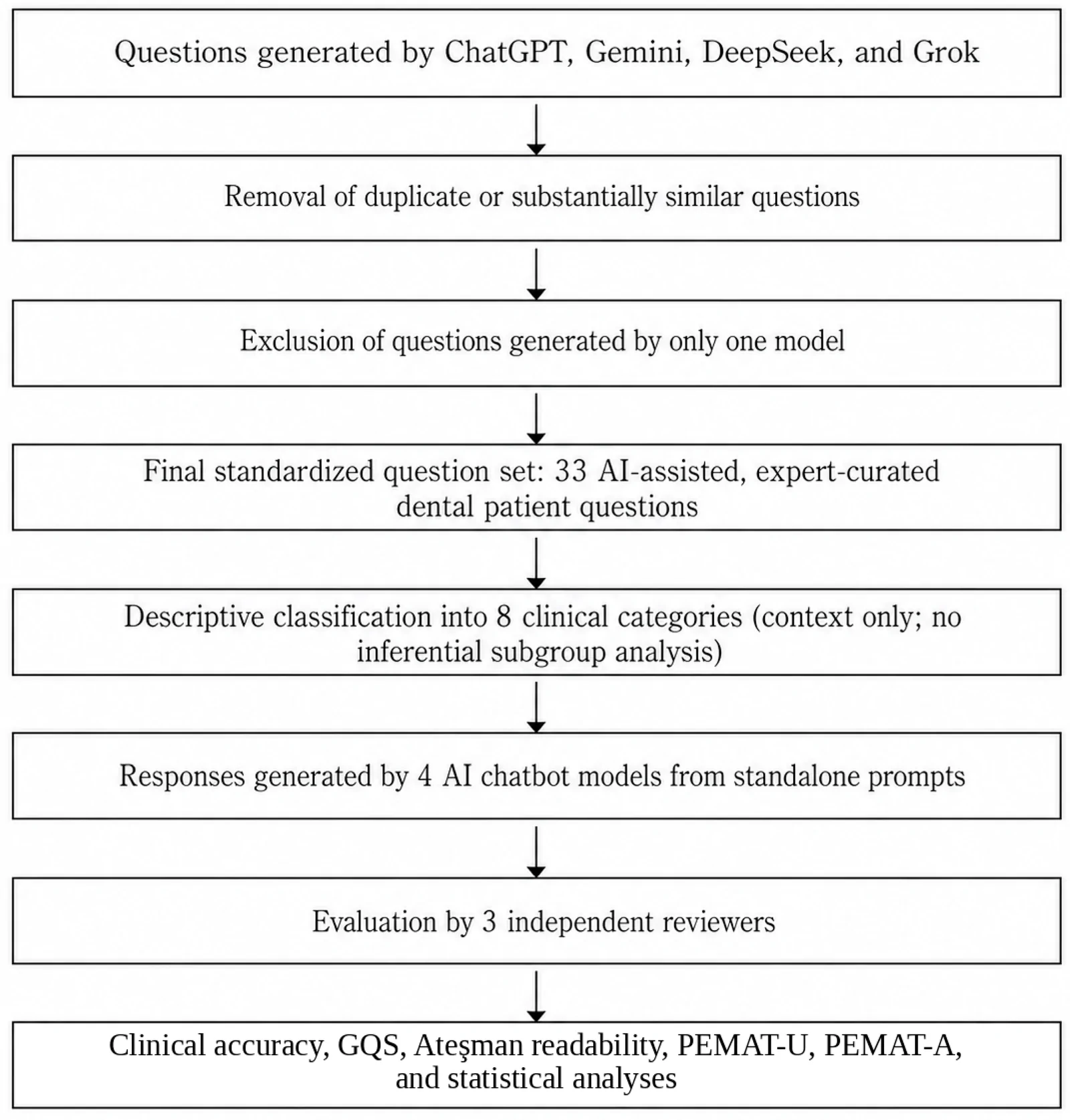
Study flowchart illustrating question selection, categorization, response generation, evaluation, and statistical analysis procedures.

This classification was retained only to describe the clinical scope of the question set and to provide contextual information regarding the types of dental concerns included in the study. The distribution was five questions in Tooth Pain and Infections and four questions in each of the other seven categories). Because each category contained only four or five questions, clinical categories were not used for primary inferential subgroup comparisons. The overall study workflow is shown in Figure 1.

### 2.2. Data Collection from AI Chatbot Models

All 33 questions were presented to each chatbot in the same order as standalone prompts, without additional instructions regarding response length, tone, reading level, citations, or formatting. No follow-up, corrective, or regeneration prompts were used; each question was submitted once in a new chat session and the first complete response was retained. Conversation history was cleared before each query. Data were collected between 15 and 25 February 2026. The model labels recorded in the study archive were ChatGPT 5.2 (OpenAI), Gemini (Google), DeepSeek V3 (DeepSeek AI), and Grok 3 (xAI). For Gemini, a more specific interface-level sub-version was not separately archived and is therefore not claimed retrospectively. All models were accessed through their public web interfaces rather than APIs, so temperature, top-p, frequency or presence penalties, and random seed could not be manually standardized. No browsing or external-search instruction was included in the prompts; however, platform-level automatic retrieval behavior was not independently logged and could not be controlled through the commercial interfaces. The full set of verbatim prompts is provided in Supplementary Table S1. Because commercial LLMs are continuously updated and generation is stochastic, the dataset represents a time-specific snapshot of the model labels and access conditions available during the February 2026 collection window.

### 2.3. Evaluation Process

All AI-generated responses were evaluated within a multidimensional framework comprising clinical accuracy, global quality, readability, understandability, and actionability. Clinical accuracy, global quality, understandability, and actionability were independently assessed by three dental professionals. Readability was calculated separately for each response using the Ateşman Readability Formula rather than being assigned as a subjective evaluator score.

All three evaluators were licensed dentists working in oral and dentomaxillofacial radiology. They comprised an oral and maxillofacial radiologist with more than 20 years of clinical experience, an oral and maxillofacial radiologist with more than 10 years of clinical experience, and an oral and maxillofacial radiology resident with at least 5 years of clinical experience. The latter two evaluators had also participated in question selection and categorization. Each evaluator scored the responses independently and did not have access to the scores assigned by the other evaluators.

For evaluator-based outcomes, the three scores were averaged for each question–model response before model comparisons because the inferential analyses were intended to represent the mean judgment of the three-evaluator panel rather than the score of any single evaluator. Averaging was not treated as a remedy for disagreement: it can stabilize a panel estimate but does not eliminate underlying rater variability. Accordingly, ICC(2,1) and ICC(2,3) were retained to quantify the reproducibility of individual ratings and of the three-rater mean, respectively, and low ICC values were treated as an explicit limitation when interpreting between-model differences. The dental question constituted the repeated-measures unit, and each model contributed 33 question-level observations per evaluation dimension.

### 2.4. Conceptual and Mathematical Operationalization of the Evaluation Dimensions

The evaluation framework was based on the premise that clinical accuracy, global quality, readability, understandability, and actionability represent related but conceptually distinct dimensions of patient-oriented health information. These dimensions were therefore evaluated separately and were not combined into a single composite performance score.

Clinical accuracy was defined as the extent to which a response was scientifically correct, clinically appropriate, sufficiently complete, and unlikely to cause harm or inappropriate patient behavior. Throughout the manuscript, this evaluator-derived outcome is termed ‘clinical accuracy’; its operational definition intentionally includes appropriateness, completeness, and safety rather than factual correctness alone. Global quality represented the overall usefulness, informational completeness, organization, flow, and educational value of the response. Readability represented surface-level linguistic complexity based on word and sentence characteristics. Understandability represented the extent to which a patient could process and explain the main information presented, whereas actionability represented the extent to which the patient could identify specific actions that could be taken on the basis of the information.

Let:

- *i* = 1, …, N denote the dental questions;
- *m* = 1, …, M denote the AI chatbot models;
- *r* = 1, …, R denote the independent evaluators.

In the present study:

*N* = 33, *M* = 4, *R* = 3.

For each question–model combination, the Global Quality Score assigned by evaluator r was denoted by G_imr_, and the clinical accuracy score was denoted by L_imr_. Both variables were scored on a scale ranging from 1 to 5.

The evaluator-averaged Global Quality Score for the response generated by model m to question i was calculated as:

$${\bar{G}}_{im}=\frac{1}{R}\sum_{r=1}^{R}G_{imr}$$

Similarly, the evaluator-averaged clinical accuracy score was calculated as:

$${\bar{L}}_{im}=\frac{1}{R}\sum_{r=1}^{R}L_{imr}$$

Because three evaluators participated in the study, these equations were operationalized as:

$${\bar{G}}_{im}=\frac{G_{im1}+G_{im2}+G_{im3}}{3}$$

$${\bar{L}}_{im}=\frac{L_{im1}+L_{im2}+L_{im3}}{3}$$

The model-level descriptive Global Quality Score was subsequently calculated across the 33 dental questions as:

$${\bar{G}}_{m}=\frac{1}{N}\sum_{i=1}^{N}{\bar{G}}_{im}$$

whereas the model-level clinical accuracy score was calculated as:

$${\bar{L}}_{m}=\frac{1}{N}\sum_{i=1}^{N}{\bar{L}}_{im}$$

Readability was calculated separately for each question–model response and did not require averaging across evaluators. PEMAT understandability and actionability percentages were initially calculated separately for each evaluator and response. The three evaluator-specific percentages were then averaged to obtain the question-level PEMAT-U and PEMAT-A scores used in the model comparisons. The mathematical calculations for readability and PEMAT scoring are described in the corresponding subsections below.

Each chatbot model was consequently represented by a multidimensional performance profile:

$$P_{m}=\left({\bar{G}}_{m},{\bar{L}}_{m},{\bar{A}}_{m},{\bar{U}}_{m},{\bar{C}}_{m}\right)$$

where ${\bar{G}}_{m}$ denotes global quality, ${\bar{L}}_{m}$ clinical accuracy, ${\bar{A}}_{m}$ readability, ${\bar{U}}_{m}$ understandability, and ${\bar{C}}_{m}$ actionability.

A single weighted composite score was not calculated because these dimensions measure different properties of patient-oriented information, and no externally validated weighting system exists for determining their relative clinical importance. Separate analysis prevented high performance in one dimension—for example, readability—from mathematically compensating for lower performance in another dimension, such as clinical accuracy or actionability.

#### 2.4.1. Clinical Accuracy Assessment

Clinical accuracy was evaluated using a predefined 5-point ordinal Likert scale [17]. Consistent with the operational definition above, the rating incorporated scientific correctness, clinical appropriateness, completeness, safety, and the potential to mislead or harm a patient; it was distinct from presentation quality, organization, or linguistic simplicity.

For the response generated by model m to question i, the clinical accuracy score assigned by evaluator r was denoted by:

L_imr_ ∈ {1, 2, 3, 4, 5}.

The scoring criteria were defined as follows:

One point: The response is inconsistent with the scientific literature, contains major factual or clinical errors, and may lead to harmful or inappropriate patient behavior.

Two points: The response contains substantial inaccuracies, clinically important deficiencies, or potentially misleading recommendations and cannot be considered clinically reliable.

Three points: The response is partially accurate but contains clinically relevant omissions, insufficient explanation, or recommendations requiring substantial clarification.

Four points: The response is largely accurate and clinically appropriate, although minor deficiencies, omissions, or imprecise statements may be present.

Five points: The response is scientifically accurate, clinically appropriate, sufficiently comprehensive, and unlikely to mislead or harm the patient.

The three evaluator-specific scores were averaged for each question–model response:

$${\bar{L}}_{im}=\frac{L_{im1}+L_{im2}+L_{im3}}{3}$$

The resulting question-level scores were used in the comparisons among the four chatbot models. Higher values indicated greater clinical accuracy under this operational definition.

#### 2.4.2. Global Quality Score

The overall quality and patient education value of the responses were evaluated using the Global Quality Score (GQS) [11]. Unlike the clinical accuracy scale, which primarily addressed scientific correctness and clinical safety, the GQS evaluated the overall presentation, organization, usefulness, and educational value of the information.

The GQS assessment considered the flow and organization of the response, completeness of the information, usefulness to patients, clarity of presentation, and overall educational value.

For the response generated by model m to question i, the GQS assigned by evaluator r was denoted by:

G_imr_ ∈ {1, 2, 3, 4, 5}.

The scores were interpreted as follows:

One point: Very poor quality; the response lacks meaningful organization or useful information and is not helpful to patients.

Two points: Poor quality; the response contains limited, insufficient, or poorly organized information and has low educational value.

Three points: Moderate quality; the response is partially useful but contains deficiencies in completeness, organization, flow, or patient-oriented explanation.

Four points: Good quality; the response is generally useful, sufficiently informative, logically organized, and appropriate for patient education, although minor limitations may remain.

Five points: Excellent quality; the response is comprehensive, well organized, clearly presented, highly useful, and provides strong educational value for patients.

The three evaluator-specific GQS values were averaged for each question–model response:

$${\bar{G}}_{im}=\frac{G_{im1}+G_{im2}+G_{im3}}{3}$$

The resulting question-level scores were used in the comparative analyses. Higher scores indicated better overall quality and greater patient education value.

#### 2.4.3. Readability Assessment

Readability was evaluated using the Ateşman Readability Formula, which was specifically developed for Turkish-language texts [14]. Because all chatbot responses were generated in Turkish, this formula was preferred over readability measures developed for English-language materials, such as the Flesch Reading Ease and Flesch–Kincaid Grade Level formulas.

Readability was treated as a response-level linguistic property rather than a subjective evaluator rating. One Ateşman score was calculated for each of the 132 question–model responses.

##### Text-Processing and Counting Procedure

To ensure reproducibility, the word, sentence, and syllable counts required for the Ateşman calculation were obtained using a deterministic rule-based computational procedure. The same frozen preprocessing, tokenization, pronunciation, and counting rules were applied to all responses without reference to model identity or the resulting readability score.

The dental questions used as prompts were excluded from the readability calculation. Lexical content generated by the chatbots, including words appearing in response headings and subheadings, was retained. Only non-linguistic formatting elements—such as markdown markers, decorative separators, emojis, bullet symbols, HTML tags, and numerals used solely to order list items—were removed. Their removal did not delete the surrounding lexical content.

Sentence boundaries were identified using sentence-final periods, question marks, and exclamation marks after protecting periods occurring in predefined abbreviations and decimal expressions. Repeated terminal punctuation was treated as a single boundary. Line breaks, bullet markers, and headings without sentence-final punctuation were not independently counted as sentence boundaries.

Words were defined as contiguous alphabetic or numeric lexical tokens separated by whitespace or punctuation. Internal apostrophes and hyphens were retained when they formed part of a single lexical expression. Numerical expressions, percentages, measurement units, abbreviations, acronyms, and non-Turkish terms occurring in the substantive response text were retained rather than excluded.

For ordinary Turkish alphabetic words, the syllable count was determined from the number of Turkish vowels (a, e, ı, i, o, ö, u, and ü). Numerical expressions were converted to their Turkish verbal forms; percentages were processed as “yüzde” followed by the relevant number; and inflected numerical expressions were processed using their contextual Turkish forms. Acronyms were read using Turkish letter names unless a conventional Turkish expansion was specified. Measurement units, common abbreviations, and non-Turkish terms were processed using a frozen corpus-specific pronunciation dictionary. Tokens not requiring a special rule were processed by the general algorithm. The frozen rules and pronunciation mappings are provided in Supplementary Method S1.

All substantive lexical tokens were therefore represented in the word and syllable counts. No headings, numerical expressions, abbreviations, acronyms, measurement units, or non-Turkish terms were excluded solely because they required a special pronunciation rule.

##### Mathematical Calculation

Let:

S_im_ denote the total number of syllables,

W_im_ denote the total number of words, and

T_im_ denote the total number of sentences

in the response generated by model m to question i.
Average word length was calculated as:

*AWL_im_* = *S_im_*/*W_im_*,

whereas average sentence length was calculated as:

*ASL_im_* = *W_im_*/*T_im_*.

The Ateşman readability score was calculated using the following equation:

*A_im_ =* 198.825 − 40.175(*S_im_/W_im_*) − 2.610(*W_im_/T_im_*).

Equivalently:

*A_im_ =* 198.825 − 40.175(*AWL_im_*) − 2.610(*ASL_im_*).

Higher scores indicated easier readability, whereas lower scores indicated greater linguistic complexity. Readability levels were classified as follows:

90–100: Very easy;

70–89: Easy;

50–69: Moderate;

30–49: Difficult;

0–29: Very difficult.

The model-level readability score was calculated as the arithmetic mean of the 33 response-level Ateşman scores:

$${\bar{A}}_{m}=\frac{1}{N}\sum_{i=1}^{N}A_{im},\;\;N=33$$

Because readability scores were calculated directly from the linguistic characteristics of each response rather than assigned by evaluators, Ateşman scores were not included in the inter-rater reliability analysis.

The complete preprocessing rules, sentence-boundary rules, tokenization procedure, frozen pronunciation dictionary, and calculation logic are provided in Supplementary Method S1 to facilitate independent replication.

##### Patient Education Materials Assessment Tool (PEMAT)

The understandability and actionability of the chatbot responses were evaluated using the Patient Education Materials Assessment Tool for printable materials (PEMAT-P) [15]. Although PEMAT-P was originally developed for printable patient education materials, each captured chatbot response was evaluated in this study as a fixed, standalone written patient-information text rather than as an ongoing conversational interaction. Accordingly, PEMAT-P was used to characterize the understandability and actionability of the recorded textual output, not the broader interactive capabilities of the chatbot.

Understandability was defined as the extent to which patients with different levels of health literacy could process and explain the principal information presented in a response. Actionability was defined as the extent to which patients could identify specific and feasible actions that could be taken on the basis of the information provided.

The standard PEMAT-P structure comprises 17 understandability items and 7 actionability items. All 132 responses were independently evaluated by the three dental evaluators using the standard item structure and item-specific applicability rules. Turkish operational wording aligned with the original PEMAT-P item content, together with scoring and applicability criteria, is provided in Supplementary Table S2.

##### Item-Level Scoring and Applicability

For each response, applicable PEMAT-P items were scored as 1 when the criterion was met and 0 when it was not met. Items were coded as N/A only when the standard PEMAT-P instructions permitted a not-applicable rating. N/A items were excluded from the denominator for that response.

Applicability was determined separately for each response. The numerical-information item (U6) was coded N/A when no numerical information was present. Items U8, U9, and U11 allow N/A for very short materials; however, none of the 132 responses met the PEMAT-P definition of a very short material, so these items remained applicable. Items U16-U18 were coded N/A for all responses because the captured outputs contained no visual aids, and U19 was coded N/A when no table was present. Only the Gemini responses to Questions 4 and 20 contained tables and were therefore scored for U19. Actionability item A24 was coded N/A for all responses because none required the reader to perform a mathematical calculation. Item A25 was coded N/A when no graph, chart, table, or diagram was present; only Gemini Questions 4 and 20 were applicable for this item.

##### Score Calculation and Aggregation

For the response generated by model m to question i and evaluated by rater r, let nU_imr_ and nA_imr_ denote the numbers of applicable understandability and actionability items, respectively. The evaluator-specific scores were calculated as:

PEMAT-*U_imr_* = 100 × (sum of scores for applicable understandability items/*nU_imr_*).

PEMAT-*A_imr_* = 100 × (sum of scores for applicable actionability items/*nA_imr_*).

Thus, N/A items did not contribute to either the numerator or denominator. The three evaluator-specific percentages were averaged to obtain the question-level PEMAT-U and PEMAT-A scores for each model, and model-level descriptive values were subsequently calculated across the 33 matched questions.

PEMAT-U and PEMAT-A were analyzed as separate continuous outcomes; no combined PEMAT score and no non-validated categorical cutoffs were used. Inter-rater reliability for the evaluator-specific PEMAT-U and PEMAT-A percentages was assessed using two-way random-effects intraclass correlation coefficients based on absolute agreement, as described below.

##### Sensitivity Analysis

A supplementary sensitivity analysis contrasted the primary standard PEMAT-P results with the earlier fixed-denominator operational scoring approach. Because that earlier checklist did not implement the standard PEMAT-P applicability procedure and was not equivalent to standard PEMAT-P scoring, it was not used for the primary inferential analysis. The sensitivity results are provided in Supplementary Table S5.

### 2.5. Statistical Analysis

Statistical analyses were conducted using IBM SPSS Statistics Version 22.0 (IBM Corp., Armonk, NY, USA) and Python 3.13.5 (NumPy 2.3.5; SciPy 1.17.0)-based statistical procedures developed for the calculation of effect sizes, bootstrap confidence intervals, and exploratory multidimensional analyses.

#### 2.5.1. Analysis Unit and Data Aggregation

The dental question was defined as the repeated-measures analysis unit. Because the same 33 questions were submitted to each of the four chatbot models, the resulting observations had a matched 33 × 4 structure for each evaluation dimension.

For evaluator-based outcomes, including clinical accuracy, Global Quality Score, PEMAT-U, and PEMAT-A, the three evaluator-specific scores were first averaged for each question–model response. Thus, each chatbot model contributed one aggregated observation per question for each outcome.

For example, the question-level clinical accuracy score was:

$${\bar{L}}_{im}=\frac{1}{R}\sum_{r=1}^{R}L_{imr},\;\;R=3$$

The corresponding model comparison was performed using the 33 values of ${\bar{L}}_{im}$ obtained for each model. The same aggregation procedure was applied to GQS, PEMAT-U, and PEMAT-A scores.

Ateşman readability was calculated directly from the linguistic characteristics of each response and therefore did not require evaluator averaging. Following completion of the deterministic counting and verification procedure, each model was represented by 33 response-level readability scores.

Descriptive statistics were presented as mean ± standard deviation and median with interquartile range, as appropriate. Total word counts were reported descriptively and were not treated as independent observations in inferential analyses.

#### 2.5.2. Omnibus Comparisons Among Chatbot Models

Differences among the four chatbot models were evaluated separately for clinical accuracy, GQS, Ateşman readability, PEMAT-U, and PEMAT-A using the Friedman test.

For each outcome, the null hypothesis was:

*H*_0_: *θ*_1_ = *θ*_2_ = *θ*_3_ = *θ*_4_,

where θ_m_ denotes the distributional location of the corresponding score for model m.

The Friedman test statistic was calculated from the within-question ranks assigned to the four chatbot models. The dental question constituted the blocking variable, with:

*N* = 33

repeated-measures blocks and:

*M* = 4

chatbot models.

The omnibus effect size was quantified using Kendall’s coefficient of concordance:

*W* = *χ*^2^*F*/[*N*(*M* − 1)],

where χ^2^F is the Friedman chi-square statistic, N is the number of matched questions, and M is the number of models.

Kendall’s W ranges from 0 to 1. A value approaching 0 indicates limited systematic separation among the model rankings, whereas a value approaching 1 indicates stronger and more consistent separation across the repeated-measures blocks.

Percentile-based 95% confidence intervals for Kendall’s W were estimated using 5000 paired bootstrap samples. During each bootstrap iteration, the 33 dental questions were sampled with replacement while preserving all four model observations belonging to each selected question.

#### 2.5.3. Pairwise Post Hoc Comparisons

When the Friedman test demonstrated a statistically significant omnibus difference, pairwise comparisons were conducted using the Wilcoxon signed-rank test.

With four chatbot models, the number of possible pairwise comparisons was:

*C*(4, 2) = 6.

To control the family-wise type I error rate, Bonferroni-adjusted *p*-values were calculated as:

*p_adjusted* = *min*(6*p_raw*, 1).

A pairwise difference was considered statistically significant when the Bonferroni-adjusted *p*-value was below 0.05.

For each comparison, question-level paired differences were defined as:

*D_i_* = *X_i_,_α_* − *X_i_,_β_*,

where X_i_,_α_ and X_i_,_β_ denote the scores obtained by models a and b, respectively, for question i. Observations with:

*D_i_* = 0

were excluded from the signed-rank calculation. Consequently, the effective sample size n_eff represented the number of non-zero paired differences.

#### 2.5.4. Pairwise Effect Sizes

Two complementary effect-size measures were calculated for each Wilcoxon comparison.

##### Standardized Wilcoxon Effect Size

The standardized Wilcoxon effect size was calculated as:

*r* = |*Z*|/√*n_eff*,

where Z is the standardized Wilcoxon signed-rank statistic and n_eff is the number of non-zero paired differences.

The absolute magnitude of r was interpreted descriptively using conventional reference values:

- Approximately 0.10: small effect;
- Approximately 0.30: moderate effect;
- Approximately 0.50 or greater: large effect.

Because the absolute value was reported, this statistic represented effect magnitude but not direction.

##### Matched-Pairs Rank-Biserial Correlation

The direction and magnitude of each paired difference were additionally quantified using the matched-pairs rank-biserial correlation:

r_rb = (W^+^ − W^−^)/(W^+^ + W^−^),

where W^+^ is the sum of the ranks corresponding to positive paired differences and W^−^ is the sum of the ranks corresponding to negative paired differences.

The rank-biserial correlation ranges from −1 to +1. For a comparison expressed as model a minus model b:

- A positive value indicates higher scores for model a;
- A negative value indicates higher scores for model b;
- A value approaching zero indicates limited directional separation.

Percentile-based 95% confidence intervals for r_rb were calculated using 5000 paired bootstrap samples. Question–model sets were resampled at the question level to preserve the matched design.

The matched-pairs rank-biserial correlation was treated as the primary pairwise effect-size measure because it conveys both the magnitude and direction of paired differences. The standardized Wilcoxon |r| was retained as a complementary, widely reported magnitude measure. Pairwise comparisons were interpreted jointly using the Bonferroni-adjusted *p*-value, effect magnitude and direction, bootstrap confidence interval, and the reliability of the underlying evaluator-derived outcome rather than statistical significance alone.

#### 2.5.5. Inter-Rater Reliability

Inter-rater reliability was evaluated separately for clinical accuracy, GQS, PEMAT-U, and PEMAT-A. Ateşman readability was not included because it was calculated from deterministic text characteristics rather than assigned by evaluators.

Reliability was assessed using a two-way random-effects intraclass correlation coefficient based on absolute agreement. Both single-measure and average-measure reliability were reported:

ICC(2,1)

represented the reliability of an individual evaluator, whereas:

ICC(2,*R*), *R* = 3,

represented the reliability of the mean score obtained from the three evaluators.

For an outcome evaluated across n response units by R evaluators, the single-measure ICC was calculated as:

ICC(2,1) = (MSB − MSE) / [MSB + (*R* − 1)MSE + *R*(MSR − MSE)/*n*],

where

- MSB denotes the mean square between response units;
- MSR denotes the mean square between evaluators;
- MSE denotes the residual mean square;
- n denotes the number of evaluated question–model responses;
- R = 3 denotes the number of evaluators.

Average-measure reliability was calculated as:

ICC(2,R) = (MSB − MSE)/[MSB + (MSR − MSE)/n].

The reliability analysis included all:

33 × 4 = 132

question–model responses for each evaluator-based outcome.

Percentile-based 95% confidence intervals for the ICC estimates were obtained using 5000 bootstrap samples. Response units were resampled with replacement while preserving the three scores assigned to each selected response.

ICC estimates were interpreted as follows:

- <0.50: poor reliability;
- 0.50–0.75: moderate reliability;
- 0.75–0.90: good reliability;
- >0.90: excellent reliability.

Both ICC(2,1) and ICC(2,3) were reported because model comparisons were based on the average of three evaluators, while the single-measure ICC provided information about the reproducibility of an individual evaluator’s assessment.

#### 2.5.6. Exploratory Multidimensional Pareto Analysis

Because the five evaluation dimensions represented different constructs and no externally validated weighting system was available, the dimensions were not combined into an arbitrarily weighted composite score. Instead, an exploratory Pareto-dominance analysis was conducted to examine whether any model demonstrated uniformly better performance across all dimensions.

Each model was represented by the performance vector:

$$P_{m}=\left({\bar{G}}_{m},{\bar{L}}_{m},{\bar{A}}_{m},{\bar{U}}_{m},{\bar{C}}_{m}\right)$$

where the components represented mean GQS, clinical accuracy, Ateşman readability, PEMAT-U, and PEMAT-A scores, respectively.

Model a was considered to dominate model b when:

$${P_{\alpha },}^{d}\geq {P_{\beta },}^{d}$$

for every dimension d, and:

$${P_{\alpha },}^{d}>{P_{\beta },}^{d}$$

for at least one dimension.

This analysis did not require numerical weighting of the five dimensions and therefore prevented superior performance in one domain from compensating for inferior performance in another.

The stability of the Pareto relationships was explored using 10,000 paired bootstrap samples. Dental questions were resampled with replacement while retaining all model scores and evaluation dimensions belonging to each selected question. The proportion of bootstrap iterations in which one model dominated another was reported as the bootstrap dominance probability.

Because this analysis was not specified before data examination, it was designated as exploratory and hypothesis-generating. Pareto dominance was used only as a descriptive method for examining weight-free multidimensional trade-offs; it is not a validated clinical performance metric and was not used to define an overall ‘best’ chatbot.

#### 2.5.7. Statistical Significance and Reporting

All hypothesis tests were two-sided. Statistical significance was defined as:

*p* < 0.05.

Exact *p*-values were reported whenever possible. For pairwise analyses, statistical significance was determined using Bonferroni-adjusted *p*-values. Omnibus and pairwise findings were reported together with their corresponding effect sizes and 95% confidence intervals.

The interpretation of model performance was not based solely on *p*-values. The magnitude and direction of the observed differences, uncertainty intervals, and inter-rater reliability were considered jointly.

## 3. Results

### 3.1. Overall Comparisons Among the Chatbot Models

A total of 132 chatbot responses, comprising 33 responses from each of the four models, were evaluated. Statistically significant differences were observed among the chatbot models for all five evaluation dimensions: Global Quality Score (GQS), clinical accuracy, Ateşman readability, PEMAT understandability, and PEMAT actionability (all *p* ≤ 0.002).

Gemini achieved the highest mean GQS, clinical accuracy, PEMAT-U, and PEMAT-A scores. For readability, the highest mean Ateşman score was observed for Grok, followed by DeepSeek, ChatGPT, and Gemini. However, pairwise analyses demonstrated that ChatGPT, DeepSeek, and Grok had comparable readability scores, and each achieved significantly higher readability than Gemini. The descriptive statistics and omnibus comparison results are summarized in Table 2.

### 3.2. Pairwise Comparisons and Effect Sizes

Bonferroni-adjusted pairwise comparisons and corresponding effect sizes are presented in Table 3. For GQS, Gemini and DeepSeek achieved significantly higher scores than Grok. A similar pattern was observed for clinical accuracy, for which Gemini and DeepSeek received significantly higher scores than Grok.

For Ateşman readability, ChatGPT, DeepSeek, and Grok each achieved significantly higher scores than Gemini. The effects were large for ChatGPT versus Gemini (|r| = 0.558), DeepSeek versus Gemini (|r| = 0.692), and Grok versus Gemini (|r| = 0.826). No significant readability differences were observed among ChatGPT, DeepSeek, and Grok.

For PEMAT-U, both ChatGPT and Gemini received significantly higher scores than Grok. For PEMAT-A, the only statistically significant pairwise difference was between Gemini and Grok, with Gemini receiving higher actionability scores.

**Figure 2:**
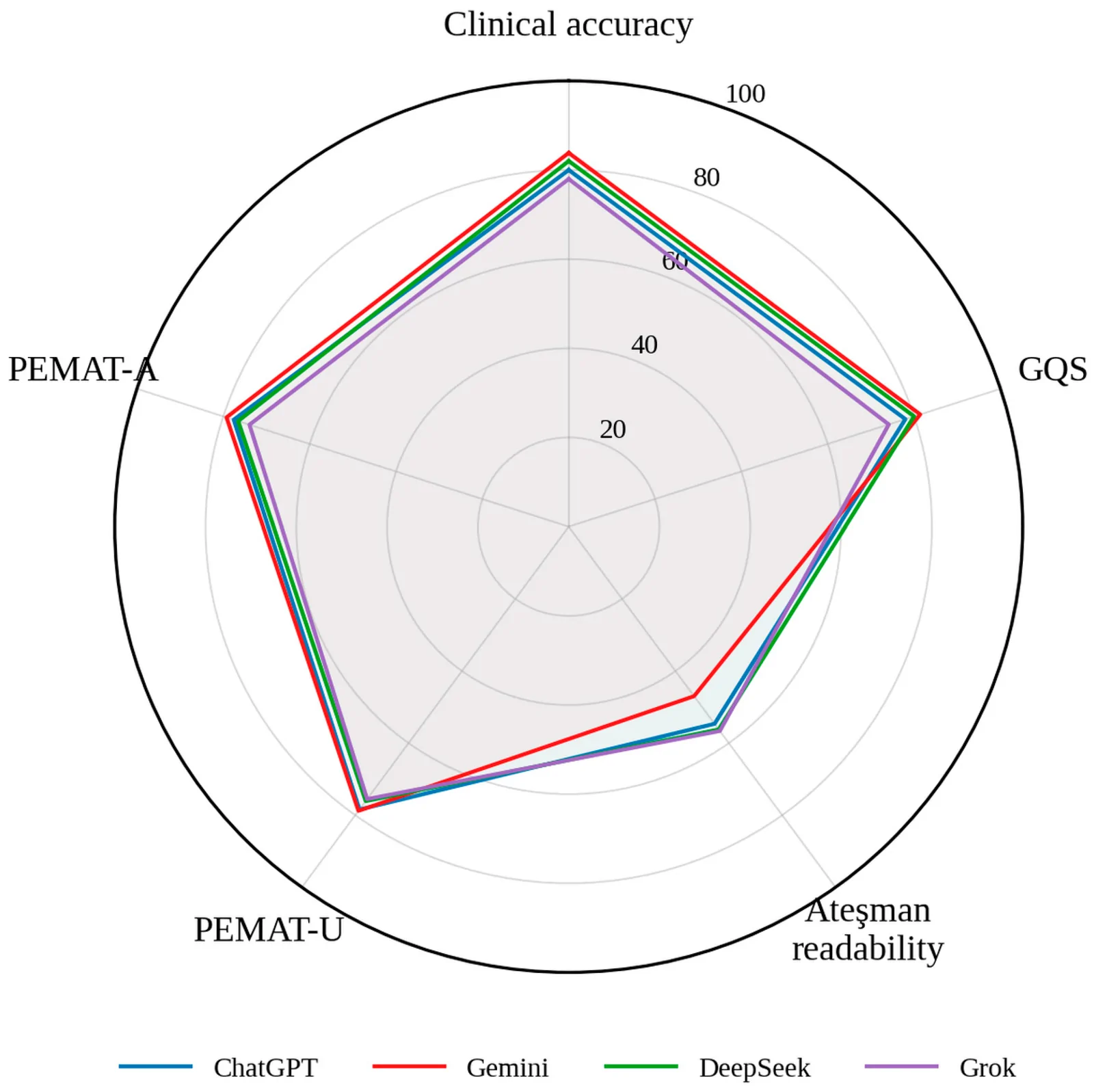
Radar chart showing the normalized descriptive performance profiles of ChatGPT, Gemini, DeepSeek, and Grok across clinical accuracy, Global Quality Score, Ateşman readability, PEMAT-U, and PEMAT-A. Scores were normalized to a 0–100 scale solely for visualization. PEMAT-U and PEMAT-A reflect standard applicability-based PEMAT-P scoring with N/A items excluded from response-specific denominators. No composite performance score was calculated.

In the sensitivity analysis, standard PEMAT-P scoring increased mean PEMAT-U values by 5.94–6.34 points and decreased mean PEMAT-A values by 14.51–16.12 points across models relative to the earlier fixed-denominator operational scores. Despite these absolute score shifts, the pattern of statistically significant between-model PEMAT contrasts was unchanged (Supplementary Table S5). The normalized descriptive performance profiles of the four chatbot models across the five evaluation dimensions are illustrated in Figure 2.

### 3.3. Omnibus Effect Sizes

The Friedman test demonstrated statistically significant differences among the four chatbot models for GQS (χ^2^(3) = 24.882, *p* < 0.001, Kendall’s W = 0.251, 95% CI: 0.147–0.418), clinical accuracy (χ^2^(3) = 21.502, *p* < 0.001, Kendall’s W = 0.217, 95% CI: 0.123–0.371), Ateşman readability (χ^2^(3) = 34.127, *p* < 0.001, Kendall’s W = 0.345, 95% CI: 0.201–0.532), PEMAT-U (χ^2^(3) = 24.754, *p* < 0.001, Kendall’s W = 0.250, 95% CI: 0.122–0.433), and PEMAT-A (χ^2^(3) = 14.568, *p* = 0.002, Kendall’s W = 0.147, 95% CI: 0.072–0.301).

### 3.4. Inter-Rater Reliability

Inter-rater reliability findings are presented in Table 4. Single-measure reliability was poor for all evaluator-based outcomes. Average-measure reliability was poor for GQS and clinical accuracy and moderate for PEMAT-U and PEMAT-A.

For the average of three evaluators, the ICC values were 0.349 for GQS, 0.486 for clinical accuracy, 0.636 for PEMAT-U, and 0.709 for PEMAT-A. The bootstrap confidence intervals were relatively wide, indicating limited precision in the reliability estimates. Therefore, statistically significant model differences, particularly for the more subjective GQS and clinical accuracy assessments, should be interpreted together with the corresponding inter-rater reliability findings.

### 3.5. Exploratory Multidimensional Pareto Analysis

The exploratory Pareto analysis demonstrated that no chatbot model dominated another across all five evaluation dimensions at the mean level. Consequently, ChatGPT, Gemini, DeepSeek, and Grok all remained within the non-dominated set.

The bootstrap probabilities of remaining non-dominated were 99.0% for Gemini, 96.4% for DeepSeek, 90.4% for ChatGPT, and 60.7% for Grok. These results indicate that although the models demonstrated different relative strengths, none could be classified as uniformly superior across clinical accuracy, global quality, readability, understandability, and actionability without imposing external weights on these dimensions (Supplementary Table S4).

Because the Pareto analysis was conducted after examination of the primary results, it was interpreted as exploratory and hypothesis-generating.

## 4. Discussion

With the increasing adoption of artificial intelligence (AI) chatbots, these systems have become widely used sources of health-related information. However, concerns remain regarding the accuracy, reliability, safety, and clinical appropriateness of the information they generate [3,11,18,19]. Although a growing number of studies have evaluated AI chatbots in dentistry, the available evidence remains heterogeneous because previous investigations have examined different dental specialties, question sets, model versions, and scoring systems [2,11,18,19,20,21]. A recent systematic review and meta-analysis also emphasized the potential of large language models for dental patient education while highlighting limitations related to accuracy, consistency, and performance in complex clinical scenarios [8].

The present study evaluated ChatGPT 5.2, Gemini, DeepSeek V3, and Grok 3 across clinical accuracy, global quality, Turkish readability, understandability, and actionability. Importantly, the study does not introduce these domains as a novel framework: closely related dental work has already combined several or all of them [11,12,13]. The contribution is instead methodological and contextual—broad Turkish patient-oriented questions across multiple dental categories, deterministic language-specific readability processing, standard PEMAT-P applicability, explicit single- and average-measure reliability, matched-question effect sizes with bootstrap uncertainty, and a clearly exploratory weight-free trade-off analysis.

Significant differences were observed among the chatbot models across all five evaluation dimensions. However, the magnitude and direction of these differences varied by outcome, and no model demonstrated uniform superiority. Gemini generally achieved higher mean scores in quality-related and patient education dimensions, whereas Grok received lower mean scores for several of these outcomes. In contrast, the readability analysis showed a different pattern: Gemini obtained significantly lower Ateşman scores than ChatGPT, DeepSeek, and Grok, while no statistically significant differences were identified among the latter three models. These findings demonstrate that model performance depends on the dimension being evaluated and cannot be adequately represented by a single overall ranking.

### 4.1. Global Quality and Clinical Accuracy

Gemini achieved the highest mean Global Quality Score and clinical accuracy score, followed closely by DeepSeek and ChatGPT. Significant pairwise differences were observed primarily between Gemini or DeepSeek and Grok. Previous research has similarly reported that Gemini can generate comprehensive, well-organized, and informative health-related responses, including patient information in orthognathic surgery and other medical contexts [18,22,23,24].

Nevertheless, the present rankings should be interpreted cautiously. Average-measure inter-rater reliability was poor for both Global Quality Score and clinical accuracy. Thus, although the Friedman and post hoc analyses identified systematic differences among the models, the three evaluators did not demonstrate strong absolute agreement when assigning scores in these relatively subjective domains. The interpretation of the intraclass correlation coefficients followed the recommendations of Koo and Li [16].

The discrepancy between statistically significant model differences and poor inter-rater reliability is methodologically important. Statistical significance indicates that the relative score distributions differed across the models, whereas the intraclass correlation coefficient evaluates whether evaluators assigned sufficiently similar absolute scores to the same responses. Consequently, a significant Friedman result does not imply that the clinical judgments underlying those scores were highly reproducible.

Subjective evaluation of AI-generated health information may be influenced by differences in evaluators’ expectations regarding completeness, acceptable clinical detail, risk communication, patient-centered language, and the clinical importance of omissions. Therefore, the observed differences in global quality and clinical accuracy should be regarded as evidence of model-specific tendencies rather than definitive proof that one chatbot is universally superior.

The present ChatGPT findings are broadly compatible with prior dental studies reporting clinically useful performance for traumatic dental injury questions, clear-aligner information, and prosthodontic patient inquiries [11,17,19]. However, the relative rankings reported across these studies differ, probably because of variations in clinical topic, model version, prompt construction, response sampling, evaluator composition, and scoring methodology [11,17,19].

### 4.2. Readability

The recalculated Ateşman scores produced a different readability pattern from the initial analysis. Grok demonstrated the highest mean score, followed by DeepSeek and ChatGPT, whereas Gemini showed the lowest mean readability. Pairwise analyses demonstrated that ChatGPT, DeepSeek, and Grok each achieved significantly higher scores than Gemini. However, no significant differences were detected among ChatGPT, DeepSeek, and Grok.

The corresponding Wilcoxon effect sizes were large for ChatGPT versus Gemini, DeepSeek versus Gemini, and Grok versus Gemini. These results indicate that the lower readability observed for Gemini was not limited to a small numerical difference. Nevertheless, the findings do not support establishing a strict readability hierarchy among ChatGPT, DeepSeek, and Grok.

Previous studies have reported model-dependent differences in the readability of AI-generated health information, including comparisons involving ChatGPT, DeepSeek, and dental chatbots [11,25]. The lower Ateşman score obtained by Gemini in the present study may reflect greater sentence length, higher syllabic complexity, or a combination of these linguistic characteristics. However, sentence length, lexical diversity, and syntactic complexity were not examined as separate inferential outcomes. These explanations should therefore be considered hypotheses rather than established mechanisms.

Readability should not be interpreted as equivalent to clinical usefulness. The Ateşman formula was developed as a Turkish-language readability index and quantifies surface-level linguistic complexity using average word and sentence characteristics [14]. It does not assess factual correctness, clinical completeness, safety, contextual adequacy, or whether a patient can implement the recommendations provided. A response may therefore be relatively easy to read but clinically incomplete, insufficiently actionable, or potentially misleading.

The present results reinforce the need to evaluate readability together with clinical accuracy, global quality, understandability, and actionability. A model with a higher readability score should not automatically be considered more appropriate for patient education, particularly when its clinical quality or actionability scores are lower. This interpretation is consistent with the conceptual distinction between readability and the understandability and actionability constructs measured by the Patient Education Materials Assessment Tool [15].

### 4.3. Understandability and Actionability

Using standard applicability-based PEMAT-P scoring, Gemini achieved the highest mean PEMAT-U and PEMAT-A scores. For understandability, Gemini and ChatGPT received significantly higher scores than Grok. For actionability, the only statistically significant pairwise difference was between Gemini and Grok. These findings suggest that a response may be linguistically readable without necessarily being structured in a manner that allows patients to understand the principal information or identify specific actions.

Readability, understandability, and actionability are related but non-interchangeable constructs. PEMAT-P was developed for printable patient education materials [15]. In the present study, it was applied to the captured chatbot responses as fixed, standalone written texts; the PEMAT findings therefore characterize the recorded outputs rather than the complete dynamic conversational experience.

The relatively higher inter-rater reliability observed for PEMAT-U and PEMAT-A compared with Global Quality Score and clinical accuracy may be related to the item-based structure of PEMAT-P. The average-measure ICCs were 0.636 for PEMAT-U and 0.709 for PEMAT-A, both within the moderate range, indicating that evaluator variability persisted despite the more structured item-level criteria [15,16].

The separate analysis of PEMAT-U and PEMAT-A was methodologically important. Combining these domains into a single score could conceal clinically meaningful differences and could allow high understandability to compensate mathematically for low actionability. The sensitivity analysis further showed that standard applicability-based scoring materially changed the absolute PEMAT percentages but preserved the pattern of significant between-model contrasts, supporting the robustness of the comparative conclusions while underscoring the importance of adhering to standard scoring rules.

### 4.4. Effect Sizes and Clinical Interpretation

The inclusion of effect sizes provides information that cannot be obtained from *p*-values alone. The omnibus Kendall’s W values indicated that model separation was strongest for readability and weakest for actionability. Pairwise Wilcoxon effect sizes and matched-pairs rank-biserial correlations further demonstrated that several statistically significant differences were also substantial in magnitude.

However, statistical effect size should not be equated directly with clinical importance. A large difference on a readability index may not produce a meaningful improvement in patient comprehension if the response remains clinically complex. Similarly, a modest numerical difference in clinical accuracy could be important if it concerns a recommendation with potential safety implications. Effect sizes should therefore be interpreted within the conceptual meaning of each outcome.

### 4.5. Exploratory Multidimensional Pareto Analysis

The exploratory Pareto analysis demonstrated that none of the four chatbot models dominated another across all five evaluation dimensions. ChatGPT, Gemini, DeepSeek, and Grok all remained within the non-dominated set at the mean level.

This finding supports a multidimensional interpretation of chatbot performance. Gemini tended to perform well in quality, accuracy, understandability, and actionability but demonstrated lower readability. Grok showed relatively favorable readability but lower performance in several quality-related domains. ChatGPT and DeepSeek displayed more balanced profiles without uniformly outperforming the other models.

The absence of a Pareto-dominant model argues against calculating a single arbitrarily weighted composite score. Such a score would require assigning numerical importance to clinical accuracy, quality, readability, understandability, and actionability without an externally validated weighting system. Different weights could produce different model rankings and could allow strong performance in one domain to compensate mathematically for poor performance in another clinically important domain.

Accordingly, model selection for patient education should depend on the intended use. A system selected to generate simple explanatory content may require a different performance profile from a system used to provide detailed preprocedural information or safety-sensitive guidance. Because the Pareto analysis was conducted as a secondary analysis after examination of the primary results, it should be regarded as exploratory and hypothesis-generating.

### 4.6. Methodological Contribution

The present study should therefore be viewed as an extension and more explicit operationalization of existing multidimensional dental chatbot research rather than as a new validated measurement framework. Its added value lies in the Turkish-language context, deterministic Ateşman implementation, standard item-specific PEMAT-P applicability, matched non-parametric effect-size reporting with bootstrap uncertainty, and simultaneous reporting of model differences and evaluator reliability. These elements address reporting and interpretive gaps that are not captured by simply combining established scales.

This approach reduces the risk of equating statistical separation with clinically robust superiority. It also makes explicit when a nominal model difference is supported by a large paired effect but rests on ratings with limited reproducibility. The exploratory Pareto component adds a descriptive view of trade-offs without imposing arbitrary weights, but it should not be interpreted as an independently validated performance index.

### 4.7. Limitations

The present study has several limitations. First, the initial question pool was generated with AI assistance and then expert-curated. Although requiring overlap across more than one model reduced model-specific idiosyncrasy, it may also have preferentially retained themes shared by the same systems later evaluated, introducing circularity and selection bias. The original model-specific question pools and item-level exclusion/merging decisions were not prospectively archived as a complete auditable source-by-source dataset, so a reliable full reconstruction cannot be made retrospectively. In addition, contemporaneous February 2026 responses were not collected for questions excluded because they appeared in only one model pool. A sensitivity analysis that adds those questions would therefore require querying later model versions and would confound question-set sensitivity with temporal/model-version change; such an analysis was not performed. Future studies should define the validation set independently of the evaluated models and prospectively retain the full candidate pool, source provenance, and exclusion reasons.

Second, the study evaluated 33 standardized Turkish questions rather than naturally occurring patient queries. Authentic queries may contain misspellings, incomplete context, ambiguous wording, emotional language, colloquial expressions, repeated follow-up turns, or highly individualized clinical information. The eight clinical categories were descriptive only (five questions in Tooth Pain and Infections and four in each remaining category), and category-specific inference was not attempted because of the small number of questions per category. Generalizability to other languages, settings, and patient populations is therefore limited.

Third, only one generation was sampled for each question–model pair, so the study characterizes the observed outputs rather than the full distribution of outputs each model could produce. The systems were accessed through public web interfaces, generation parameters were not controllable, and platform-level automatic retrieval behavior was not independently logged. The study archive recorded the labels ChatGPT 5.2, Gemini, DeepSeek V3, and Grok 3, but did not preserve a more specific Gemini sub-version. Commercial models are continuously updated; therefore, identical prompts may yield different outputs at another time or under different interface settings. The findings are a time-specific snapshot of 15–25 February 2026, and repeated-generation designs would be required to quantify within-model stochastic variability.

Fourth, GQS and clinical accuracy demonstrated poor inter-rater reliability. Averaging the three evaluator scores represented the panel mean but did not remove disagreement among raters. No prospectively defined question-specific reference answers, key-information checklists, formal model-blinding procedure, or independent adjudicator were used for the original clinical-accuracy ratings. Constructing such a reference standard only after seeing the model outputs could itself introduce hindsight bias; therefore, a post hoc error-frequency analysis was not retrofitted into the present dataset. These limitations reduce confidence in definitive clinical ranking despite statistically significant model differences. Future studies should prospectively define authoritative reference answers and essential-information checklists, distinguish major factual errors, important omissions and potentially harmful advice, calibrate and blind raters where feasible, and use independent adjudication for disagreements.

Fifth, the Ateşman formula measures linguistic complexity but does not directly measure patient comprehension [14]. Actual patients were not asked to read, interpret, recall, or act on the responses. Moreover, the models may not have equivalent Turkish linguistic competence, lexical diversity, or syntactic flexibility; a higher Ateşman score can therefore reflect simpler language generation as well as deliberate patient-centered simplification. Accordingly, higher readability, PEMAT-U, or PEMAT-A scores should not be interpreted as direct evidence of superior real-world patient comprehension or outcomes [15].

Sixth, PEMAT-P was originally developed for conventional printable patient education materials rather than dynamic conversational AI. Although the chatbot responses were captured and evaluated as fixed standalone written texts, this application does not establish that PEMAT-P has been specifically validated for interactive chatbot exchanges. The PEMAT findings should therefore be interpreted as properties of the recorded textual responses rather than of the complete conversational system.

Seventh, the deterministic text-processing procedure required predefined rules for numerical expressions, abbreviations, acronyms, measurement units, and non-Turkish terms. Although the same frozen rules were applied to all responses and the complete procedure was documented to support reproducibility, alternative tokenization or pronunciation conventions could produce slightly different readability values.

Finally, the Pareto analysis was conducted as a secondary exploratory analysis after examination of the primary results. Its findings should therefore be considered hypothesis-generating and require validation in larger, prospectively designed studies.

### 4.8. Strengths

Despite these limitations, this study has several strengths. All models received identical standalone prompts during a single predefined collection window, and the same 33 questions served as matched analysis units. The study did not rely on a single overall score; instead, it separated clinical accuracy, quality, Turkish readability, understandability, and actionability and reported effect magnitude, uncertainty, and evaluator reliability. The contribution is reproducibility-oriented reporting and language-specific operationalization rather than creation of a new validated instrument. The complete deterministic readability procedure, standard PEMAT-P scoring rules, pairwise analyses, and exploratory Pareto results are provided to facilitate independent scrutiny and replication.

## 5. Conclusions

The evaluated AI chatbots demonstrated distinct performance profiles across clinical accuracy, global quality, readability, understandability, and actionability. Gemini tended to achieve higher scores in quality-related and patient education dimensions but obtained significantly lower readability scores than ChatGPT, DeepSeek, and Grok. No significant readability differences were observed among the latter three models.

The poor inter-rater reliability observed for the subjective quality and clinical accuracy assessments limits the ability to establish a definitive model hierarchy. Furthermore, the exploratory Pareto analysis demonstrated that no model was uniformly superior across all five dimensions. These findings support a multidimensional approach to evaluating AI-generated dental information rather than reliance on a single score or model ranking.

AI chatbots may serve as supplementary tools for dental patient education, but their responses should not replace professional consultation or clinical decision-making. These findings characterize the sampled outputs generated by the evaluated model versions under the specific access conditions used during 15–25 February 2026 and should not be interpreted as fixed properties of continuously evolving commercial AI systems. Future studies should evaluate larger sets of authentic patient-generated questions, incorporate direct patient comprehension outcomes, apply calibrated multidisciplinary evaluator panels, and examine additional languages and clinical settings.

## Figures and Tables

**Table 1:** Methodological comparison with closely related multidimensional dental chatbot studies.

Study	Clinical Material and Models	Evaluation Domains/Instruments	Reliability and Analysis	Methodological Distinction
Özcivelek and Özcan [11]	35 patient questions on dental/maxillofacial prostheses; DeepSeek-R1, ChatGPT-o1, ChatGPT-4, Dental GPT	5-point accuracy; GQS; FRE/FKGL readability; PEMAT-P understandability/actionability	2 prosthodontists; Cohen κ; Kruskal–Wallis/ANOVA with post hoc tests	Same five broad outcome domains; English/prosthodontic focus; AI-assisted question pool with explicit exclusions.
Sivaramakrishnan et al. [12]	4 common dental patient education scenarios; ChatGPT-4.0, Claude 3.5 Sonnet, Gemini 1.5 Flash, Llama 3.1-405B	Flesch readability; PEMAT understandability/actionability	5 dental professionals; Fleiss κ	Patient education material focus with strong reliability reporting; no separate clinical-accuracy or GQS outcome.
Wu et al. [13]	40 implantology questions across 8 themes plus 5 complex cases; 5 contemporary LLMs including ChatGPT, DeepSeek, Grok, and Gemini	Five expert-scored performance dimensions	3 senior experts; ICC; Friedman; mixed-effects models; PCA	Specialty-specific professional/complex-case tasks; API parameters standardized; multivariate analysis.
Present study	33 Turkish patient questions across 8 descriptive dental categories; ChatGPT 5.2, Gemini, DeepSeek V3, Grok 3	Clinical accuracy; GQS; deterministic Ateşman readability; standard PEMAT-P understandability/actionability	3 dental evaluators; ICC(2,1)/ICC(2,3); Friedman/Wilcoxon; effect sizes and paired bootstrap CIs	Turkish-language patient context; item-specific PEMAT applicability; reliability-aware inference; exploratory Pareto trade-off analysis.

**Table 2:** Descriptive statistics, Friedman test results, omnibus effect sizes, and bootstrap confidence intervals for the five evaluation dimensions.

Outcome	ChatGPT	Gemini	DeepSeek	Grok	χ^2^	*p* Value	Kendall’s W	Bootstrap 95% CI
**GQS**	3.90 ± 0.40 ^ab^	4.07 ± 0.22 ^a^	4.00 ± 0.63 ^a^	3.71 ± 0.38 ^b^	24.882	<0.001	0.251	0.147–0.418
**Clinical accuracy**	4.00 ± 0.35 ^ab^	4.19 ± 0.24 ^a^	4.10 ± 0.62 ^a^	3.90 ± 0.39 ^b^	21.502	<0.001	0.217	0.123–0.371
**Ateşman readability**	54.65 ± 12.29 ^a^	46.98 ± 7.74 ^b^	56.22 ± 7.21 ^a^	56.64 ± 7.58 ^a^	34.127	<0.001	0.345	0.201–0.532
PEMAT-U	78.43 ± 2.63 ^a^	78.79 ± 2.33 ^a^	76.07 ± 12.42 ^ab^	75.54 ± 5.90 ^b^	24.754	<0.001	0.250	0.122–0.433
PEMAT-A	77.58 ± 5.22 ^ab^	79.26 ± 2.94 ^a^	76.57 ± 14.15 ^ab^	73.94 ± 10.98 ^b^	14.568	0.002	0.147	0.072–0.301

Data are presented as mean ± standard deviation. Overall comparisons were performed using the Friedman test. Kendall’s W represents the omnibus effect size, and its 95% confidence intervals were estimated using 5000 paired bootstrap samples. Within each row, values that do not share a superscript letter differ significantly in Bonferroni-adjusted Wilcoxon signed-rank comparisons. GQS: Global Quality Score; PEMAT-U: Patient Education Materials Assessment Tool—Understandability; PEMAT-A: Patient Education Materials Assessment Tool—Actionability; CI: confidence interval. PEMAT-U and PEMAT-A percentages were calculated using response-specific applicable-item denominators; items coded N/A were excluded from both numerator and denominator.

**Table 3:** Statistically significant pairwise comparisons and corresponding effect sizes.

Outcome	Comparison	Adjusted *p* Value	|r|	Rank-Biserial r	Bootstrap 95% CI
GQS	Gemini–Grok	<0.001	0.800	0.890	0.649–1.000
GQS	DeepSeek–Grok	0.005	0.653	0.741	0.396–0.978
Clinical accuracy	Gemini–Grok	<0.001	0.781	0.880	0.680–1.000
Clinical accuracy	DeepSeek–Grok	0.013	0.655	0.739	0.363–1.000
Ateşman readability	ChatGPT–Gemini	0.008	0.558	0.640	0.305–0.914
Ateşman readability	DeepSeek–Gemini	<0.001	0.692	0.793	0.537–1.000
Ateşman readability	Grok–Gemini	<0.001	0.826	0.947	0.832–1.000
PEMAT-U	ChatGPT–Grok	0.029	0.705	0.794	0.364–1.000
PEMAT-U	Gemini–Grok	0.002	0.740	0.837	0.547–1.000
PEMAT-A	Gemini–Grok	0.012	0.890	1.000	1.000–1.000

Only comparisons that remained statistically significant after Bonferroni adjustment are presented. The standardized Wilcoxon effect size was calculated as *|r| = |Z|/√n_eff*. Rank-biserial correlations are oriented so that positive values indicate higher scores for the first-listed model. Confidence intervals for rank-biserial correlations were estimated using 5000 paired bootstrap samples. The complete results for all pairwise comparisons are provided in Supplementary Table S3.

**Table 4:** Inter-rater reliability estimates with bootstrap confidence intervals.

Outcome	ICC Type	ICC Value	Bootstrap 95% CI	Interpretation
GQS	ICC(2,1)	0.152	−0.031–0.356	Poor
GQS	ICC(2,3)	0.349	−0.100–0.624	Poor
Clinical accuracy	ICC(2,1)	0.240	−0.004–0.474	Poor
Clinical accuracy	ICC(2,3)	0.486	−0.011–0.730	Poor
PEMAT-U	ICC(2,1)	0.368	−0.021–0.639	Poor
PEMAT-U	ICC(2,3)	0.636	−0.067–0.841	Moderate
PEMAT-A	ICC(2,1)	0.449	−0.021–0.715	Poor
PEMAT-A	ICC(2,3)	0.709	−0.066–0.883	Moderate

ICCs were calculated using a two-way random-effects model based on absolute agreement. ICC(2,1) represents single-evaluator reliability, whereas ICC(2,3) represents the reliability of the mean of three evaluators. Confidence intervals were obtained using 5000 bootstrap samples. Negative confidence limits indicate that the reliability estimate was imprecise and should not be interpreted as evidence of negative reliability.

## Data Availability

The data supporting the findings of this study are available from the corresponding author upon reasonable request.

## Supplementary Materials

The following supporting information can be downloaded at: https://www.mdpi.com/article/10.3390/diagnostics16203430/s1, Supplementary Method S1: Deterministic Text-Processing and Ateşman Readability Calculation Algorithm; Table S1: Verbatim dental patient questions submitted to each AI chatbot model; Table S2: Standard PEMAT-P Item Structure, Turkish Operational Wording, and Applicability Rules Used in the Revised Analysis; Table S3: Complete pairwise comparisons among ChatGPT, Gemini, DeepSeek, and Grok across the five evaluation dimensions; Table S4: Exploratory multidimensional Pareto analysis across clinical accuracy, global quality, readability, understandability, and actionability; Table S5: Sensitivity analysis comparing standard PEMAT-P scoring with the earlier fixed-denominator operational scoring approach.
