# Supplementary material for: Quality, Readability, Understandability, and Actionability of AI Chatbots’ Responses to Dental Patient Questions: A Comparative Evaluation
Source: Diagnostics (Basel). 2026 Oct 21;16(20):3430. doi: 10.3390/diagnostics16203430 (PMC13703409; doi:10.3390/diagnostics16203430)
Supplement: Supplementary file 1 [file diagnostics-16-03430-s001.zip › Supplementary Method S1 Atesman Algorithm Revised.pdf]

# Supplementary Method S1

*Deterministic Text-Processing and Ateşman Readability Calculation Algorithm*

*Supplement to: Multidimensional Evaluation of AI Chatbot Responses to Dental Patient Questions*

## S1.1. Purpose and Scope

This supplementary method describes the deterministic rule-based procedure used to calculate the word, sentence, and syllable counts required for the Ateşman readability score. The same frozen rules were applied to all 132 Turkish-language chatbot responses. The procedure retained all substantive lexical content while excluding only non-linguistic formatting elements.

The patient questions used as prompts were excluded because the analysis concerned the chatbot-generated responses. Headings and subheadings generated within the responses were retained as lexical content. Numerical expressions, percentages, measurement units, abbreviations, acronyms, and non-Turkish terms were also retained and processed using predefined verbalization or pronunciation rules.

## S1.2. Mathematical Definitions

For the response generated by model m to question i:

- $S_{im}$ denotes the total number of syllables.
- $W_{im}$ denotes the total number of words.
- $T_{im}$ denotes the total number of sentences.

Average word length and average sentence length were calculated as:

$$AWL_{im} = S_{im} / W_{im}$$

$$ASL_{im} = W_{im} / T_{im}$$

The Ateşman readability score was calculated as:

$$A_{im} = 198.825 - 40.175(S_{im} / W_{im}) - 2.610(W_{im} / T_{im})$$

Higher scores indicated easier readability. Scores were interpreted as 90–100, very easy; 70–89, easy; 50–69, moderate; 30–49, difficult; and 0–29, very difficult.

## S1.3. Frozen Text-Processing and Counting Rules

| Component | Deterministic rule | Rationale | Inclusion status |
|---|---|---|---|
| Source text | The patient prompt was excluded. The complete chatbot-generated response, including response headings and subheadings, was retained. | The outcome measures readability of the generated answer rather than the question. | Response lexical content included. |
| Unicode normalization | Text was normalized to Unicode NFC form. | Prevents visually identical characters from being processed differently. | No lexical exclusion. |
| Formatting elements | Markdown markers, decorative separators, emojis, bullet symbols, HTML tags, and numerals used solely as list-order markers were removed without deleting surrounding words. | Formatting symbols do not represent patient-readable lexical content. | Excluded only when non-linguistic. |
| Sentence boundaries | Periods, question marks, and exclamation marks defined sentence boundaries after abbreviation and decimal protection. Repeated terminal marks were treated as one | Provides a reproducible sentence-segmentation rule. | All substantive sentences included. |

| | | | |
|---|---|---|---|
| | boundary. | | |
| Line breaks and headings | Line breaks, bullet markers, and headings without terminal punctuation were not counted as independent sentence boundaries; their words remained in the word and syllable counts. | Distinguishes visual layout from sentence termination. | Lexical content included. |
| Word tokens | Contiguous alphabetic and numeric lexical expressions were counted as words. Internal apostrophes and hyphens were retained when part of one expression. | Provides consistent tokenization. | Included. |
| Ordinary Turkish words | Syllable count equaled the number of Turkish vowels: a, e, ı, i, o, ö, u, ü. | Operationalizes the standard Turkish syllable principle. | Included. |
| Numerical expressions | Numbers were counted as lexical tokens and converted to their Turkish verbal forms for syllable counting. Decimal and inflected forms were handled by frozen rules. | Digits do not directly encode pronunciation. | Included. |
| Percentages | Percent expressions were processed as "yüzde" followed by the Turkish verbal form of the number and any contextual suffix. | Reflects how the expression is read in Turkish. | Included. |
| Acronyms | Acronyms were counted as one lexical token and read using Turkish letter names unless a conventional Turkish expansion was specified in the frozen dictionary. | Written vowel count may not represent acronym pronunciation. | Included. |
| Abbreviations and units | Common abbreviations and measurement units were counted as one lexical token and processed using frozen Turkish expansions or pronunciations. | Ensures consistent handling of forms such as Dr., mm, and TL. | Included. |
| Non-Turkish terms | Non-Turkish terms were counted as lexical tokens and processed using a frozen corpus-specific pronunciation mapping. | The Turkish written-vowel rule may not represent their contextual pronunciation. | Included. |
| Invalid responses | A response with W=0 or T=0 was not scored and triggered an error. | Prevents division by zero and invalid values. | Not applicable to the analyzed data. |

## S1.4. Frozen Pronunciation Dictionary

A corpus-specific dictionary was frozen before the definitive analysis run. It contained the verbalization or pronunciation rule for every nonstandard token class observed in the response corpus, including numerical suffixes, percentages, abbreviations, acronyms, measurement units, and non-Turkish terms. The dictionary was applied identically across models and questions. No token was included or excluded according to the model that generated it or according to its effect on the resulting readability score.

Examples of the frozen handling rules included:

- Numerals: converted to Turkish words (e.g., 20 → "yirmi").
- Percentages: read as "yüzde" plus the number (e.g., %20 → "yüzde yirmi").
- Inflected numerals: converted to the corresponding contextual Turkish form (e.g., 20'lik → "yirmilik").
- Acronyms: read using Turkish letter names or a predefined conventional expansion (e.g., CBCT → "ce-be-ce-te").
- Measurement units and abbreviations: read using a frozen Turkish expansion (e.g., mm → "milimetre"; Dr. → "doktor").

- Non-Turkish terms: assigned a frozen contextual pronunciation mapping rather than being removed from the analysis.

The complete token-level dictionary used in the final computation is retained in the analysis archive as an auditable machine-readable file.

## S1.5. Algorithmic Workflow

1. Import the original chatbot-generated response text.
2. Exclude the patient prompt and any appended calculation notes not belonging to the chatbot response.
3. Normalize Unicode characters.
4. Remove only non-linguistic formatting elements and list-order markers.
5. Protect periods in predefined abbreviations and decimal expressions.
6. Identify sentence boundaries and calculate $T_{im}$.
7. Tokenize the retained substantive text and calculate $W_{im}$.
8. Apply the Turkish-vowel rule to ordinary Turkish words.
9. Apply the frozen verbalization or pronunciation dictionary to numbers, percentages, acronyms, abbreviations, units, and non-Turkish terms.
10. Sum syllables to calculate $S_{im}$ and then calculate $AWL_{im}$, $ASL_{im}$, and $A_{im}$.
11. Export the response-level counts, derived values, and applied token-rule classes.

## S1.6. Deterministic Rule Freezing and Auditability

All general rules and corpus-specific mappings were frozen before the definitive full-dataset run. The final run was then performed from the beginning for all 132 responses using the same rule set. No model-specific rule and no post hoc adjustment based on statistical significance were permitted.

The analysis archive contains the original response texts, frozen general rules, frozen pronunciation dictionary, response-level word/sentence/syllable counts, and final Ateşman dataset. This structure enables independent replication and audit of every derived readability score.

## S1.7. Required Output Dataset

| Variable | Description | Type |
|---|---|---|
| response_id | Unique response identifier | Text |
| question_id | Question number, 1–33 | Integer |
| model | ChatGPT, Gemini, DeepSeek, or Grok | Categorical |
| word_count | Final $W_{im}$ | Integer |
| sentence_count | Final $T_{im}$ | Integer |
| syllable_count | Final $S_{im}$ | Integer |
| average_word_length | $S_{im}/W_{im}$ | Decimal |
| average_sentence_length | $W_{im}/T_{im}$ | Decimal |
| atesman_score | Final Ateşman readability score | Decimal |
| special_token_rules | Frozen rule classes applied within the response | Text |
| analysis_version | Identifier for the frozen algorithm and dictionary | Text |

## S1.8. Implementation Logic

The following pseudocode summarizes the definitive processing logic:

```
clean_text = remove_nonlinguistic_formatting(response_text)
sentence_count = count_terminal_boundaries(clean_text, protected_abbreviations, protected_decimals)
word_tokens = tokenize_substantive_lexical_content(clean_text)
for token in word_tokens:
    if token in frozen_pronunciation_dictionary: syllables += dictionary[token]
    elif token is numerical: syllables += syllables(Turkish_verbalization(token))
    else: syllables += count_Turkish_vowels(token)
```

```
atesman = 198.825 - 40.175*(syllables/words) - 2.610*(words/sentences)
```

## S1.9. Quality-Control Checks

- Every response must have $W_{im} > 0$ and $T_{im} > 0$.
- Word, sentence, and syllable counts must be integers.
- Every nonstandard token must resolve to a documented frozen rule.
- Average word length must equal $S_{im}/W_{im}$ and average sentence length must equal $W_{im}/T_{im}$.
- The stored Ateşman score must reproduce the stated formula within a predefined numerical tolerance.
- The same algorithm version and pronunciation dictionary must be used for all 132 responses.
- Any rule change requires reprocessing the complete dataset from the beginning.

## S1.10. Recommended Reporting Statement for the Main Manuscript

*"Word, sentence, and syllable counts were generated using a deterministic rule-based Python procedure. Only non-linguistic formatting elements and list-order markers were excluded. Lexical content in response headings and subheadings was retained. Numerical expressions, percentages, abbreviations, acronyms, measurement units, and non-Turkish terms were included and processed using frozen Turkish verbalization or corpus-specific pronunciation rules. The same rules were applied to all 132 responses, and the complete procedure is provided in Supplementary Method S1."*
