# Supplementary material for: Quality, Readability, Understandability, and Actionability of AI Chatbots’ Responses to Dental Patient Questions: A Comparative Evaluation
Source: Diagnostics (Basel). 2026 Oct 21;16(20):3430. doi: 10.3390/diagnostics16203430 (PMC13703409; doi:10.3390/diagnostics16203430)
Supplement: Supplementary file 1 [file diagnostics-16-03430-s001.zip › Supplementary Table S2 PEMAT Revised.pdf]

Supplementary Table S2.

Standard PEMAT-P Item Structure, Turkish Operational Wording, and Applicability Rules Used in the Revised Analysis

All 132 chatbot responses were evaluated using the standard PEMAT-P structure (17 understandability items and 7 actionability items). The Turkish operational wording was aligned with the content of the original PEMAT-P items. Items were scored 1 when the criterion was met, 0 when it was not met, and N/A only when permitted by the standard PEMAT-P applicability rules. N/A items were excluded from the response-specific denominator.

Domain	Item	Turkish operational wording	English item meaning	Score = 1	Score = 0	Applicability rule
Understandability	U1	Materyalin amacı tamamen açık mı?	The material makes its purpose evident.	Criterion met	Criterion not met	Applicable to all responses
Understandability	U2	Materyal, amacından uzaklaştıran bilgi veya içerik içermiyor mu?	The material does not include content that distracts from its purpose.	Criterion met	Criterion not met	Applicable to all responses
Understandability	U3	Materyalde yaygın ve günlük dil kullanılmış mı?	Common, everyday language is used.	Criterion met	Criterion not met	Applicable to all responses
Understandability	U4	Tıbbi terimler yalnızca gerektiğinde kullanılmış ve kullanıldığında açıklanmış mı?	Medical terms are used only when needed and are explained.	Criterion met	Criterion not met	Applicable to all responses
Understandability	U5	Materyalde ağırlıklı olarak etken anlatım kullanılmış mı?	The material predominantly uses active voice.	Criterion met	Criterion not met	Applicable to all responses
Understandability	U6	Materyalde yer alan sayılar açık ve kolay anlaşılır mı?	Numbers, when present, are clear and easy to understand.	Criterion met	Criterion not met	N/A when no numerical information is present
Understandability	U7	Materyal kullanıcıdan herhangi bir hesaplama yapmasını beklemiyor mu?	The material does not require the user to perform calculations.	Criterion met	Criterion not met	Applicable to all responses
Understandability	U8	Bilgiler kısa ve yönetilebilir bölümlere ayrılmış mı?	Information is divided into short, manageable sections.	Criterion met	Criterion not met	N/A for very short material
Understandability	U9	Bölümlerde bilgilendirici başlıklar kullanılmış mı?	Sections use informative headings.	Criterion met	Criterion not met	N/A for very short material
Understandability	U10	Bilgiler mantıklı bir sırayla sunulmuş mu?	Information is presented in a logical sequence.	Criterion met	Criterion not met	Applicable to all responses
Understandability	U11	Materyal ana noktaları özetliyor mu?	The material includes a summary of the main points.	Criterion met	Criterion not met	N/A for very short material
Understandability	U12	Ana noktalara dikkat çekmek için görsel ipuçları (madde işareti, kalın yazı, kutu, ok, vurgu vb.) kullanılmış mı?	Visual cues such as bullets, bolding, boxes, arrows, or emphasis highlight key points.	Criterion met	Criterion not met	Applicable to all responses
Understandability	U15	Görsel yardımcılar, içeriği daha kolay anlaşılır hale getirebileceği durumlarda kullanılmış mı?	Visual aids are used when they could make the content easier to understand.	Criterion met	Criterion not met	Applicable to all responses
Understandability	U16	Görsel yardımcılar içeriği destekliyor ve dikkat dağıtmıyor mu?	Visual aids reinforce the content rather than distract from it.	Criterion met	Criterion not met	N/A when no visual aids are present
Understandability	U17	Görsel yardımcıların açık başlıkları veya açıklamaları var mı?	Visual aids have clear titles or captions.	Criterion met	Criterion not met	N/A when no visual aids are present

Domain	Item	Turkish operational wording	English item meaning	Score = 1	Score = 0	Applicability rule
Understandability	U18	İllüstrasyonlar ve fotoğraflar açık ve karmaşık olmayan nitelikte mi?	Illustrations and photographs are clear and uncomplicated.	Criterion met	Criterion not met	N/A when no visual aids are present
Understandability	U19	Tablolar basit mi ve satır/sütun başlıkları kısa ve açık mı?	Tables are simple and use brief, clear row/column headings.	Criterion met	Criterion not met	N/A when no table is present
Actionability	A20	Materyal, kullanıcının yapabileceği en az bir eylemi açıkça tanımlıyor mu?	The material clearly identifies at least one action the user can take.	Criterion met	Criterion not met	Applicable to all responses
Actionability	A21	Eylemler açıklanırken kullanıcıya doğrudan hitap ediliyor mu?	The material addresses the user directly when describing actions.	Criterion met	Criterion not met	Applicable to all responses
Actionability	A22	Eylemler yönetilebilir ve açık adımlara ayrılmış mı?	Actions are broken into manageable, explicit steps.	Criterion met	Criterion not met	Applicable to all responses
Actionability	A23	Kullanıcının eyleme geçmesine yardımcı olabileceği durumlarda somut bir araç (örn. kontrol listesi veya planlayıcı) sağlanmış mı?	A tangible tool is provided when it could help the user take action.	Criterion met	Criterion not met	Applicable to all responses
Actionability	A24	Hesaplama gerekiyorsa, nasıl yapılacağına ilişkin basit talimat veya örnek verilmiş mi?	When calculation is required, simple instructions or an example are provided.	Criterion met	Criterion not met	N/A when no calculation is required
Actionability	A25	Grafik, şema, tablo veya diyagramların eyleme geçmek için nasıl kullanılacağı açıklanmış mı?	When graphs, charts, tables, or diagrams are present, their use for taking action is explained.	Criterion met	Criterion not met	N/A when none of these elements are present
Actionability	A26	Talimatlara göre hareket etmeyi kolaylaştırabileceği durumlarda görsel yardımcılar kullanılmış mı?	Visual aids are used when they could make acting on instructions easier.	Criterion met	Criterion not met	Applicable to all responses

Corpus-specific applicability: U6 was N/A for 8 question-model responses with no numerical information. No response met the PEMAT-P definition of a very short material; therefore U8, U9, and U11 remained applicable. U16-U18 were N/A for all responses because no visual aids were present. U19 and A25 were applicable only to Gemini Questions 4 and 20 because those two responses contained tables. A24 was N/A for all responses because none required a mathematical calculation.

Scoring note: PEMAT-U and PEMAT-A percentages were calculated as points earned divided by the number of applicable items, multiplied by 100. N/A items were excluded from both numerator and denominator. The three evaluator-specific percentages were averaged for each question-model response.
