# Supplementary material for: Quality, Readability, Understandability, and Actionability of AI Chatbots’ Responses to Dental Patient Questions: A Comparative Evaluation
Source: Diagnostics (Basel). 2026 Oct 21;16(20):3430. doi: 10.3390/diagnostics16203430 (PMC13703409; doi:10.3390/diagnostics16203430)
Supplement: Supplementary file 1 [file diagnostics-16-03430-s001.zip › Supplementary Table S5 PEMAT Sensitivity.pdf]

Supplementary Table S5.

Sensitivity analysis comparing standard PEMAT-P scoring with the earlier fixed-denominator operational scoring approach

The standard PEMAT-P analysis is the primary analysis. This sensitivity table is provided to show how the revised applicability-based scoring affected absolute PEMAT percentages and whether the between-model conclusions were robust to the scoring approach.

Domain	Model	Standard PEMAT-P mean ± SD	Earlier operational mean ± SD	Mean change
PEMAT-U	ChatGPT	78.43 ± 2.63	72.09 ± 3.22	+6.34
PEMAT-U	Gemini	78.79 ± 2.33	72.81 ± 3.86	+5.98
PEMAT-U	DeepSeek	76.07 ± 12.42	70.13 ± 12.10	+5.94
PEMAT-U	Grok	75.54 ± 5.90	69.29 ± 6.37	+6.24
PEMAT-A	ChatGPT	77.58 ± 5.22	93.64 ± 6.49	−16.07
PEMAT-A	Gemini	79.26 ± 2.94	95.38 ± 5.26	−16.12
PEMAT-A	DeepSeek	76.57 ± 14.15	92.35 ± 17.41	−15.78
PEMAT-A	Grok	73.94 ± 10.98	88.45 ± 14.96	−14.51

The absolute percentages changed materially after standard applicability-based scoring; however, the statistically significant between-model pattern was preserved. For PEMAT-U, ChatGPT and Gemini remained significantly higher than Grok. For PEMAT-A, Gemini remained significantly higher than Grok. The earlier operational scores are shown only as a sensitivity comparison and were not used for the primary revised inference.
