# Supplementary material for: Quality, Readability, Understandability, and Actionability of AI Chatbots’ Responses to Dental Patient Questions: A Comparative Evaluation
Source: Diagnostics (Basel). 2026 Oct 21;16(20):3430. doi: 10.3390/diagnostics16203430 (PMC13703409; doi:10.3390/diagnostics16203430)
Supplement: Supplementary file 1 [file diagnostics-16-03430-s001.zip › Supplementary Tables S3-S4 PEMAT Revised.pdf]

Supplementary Tables S3 and S4

Complete pairwise effect-size results and exploratory Pareto analysis

Supplementary Table S3

Complete pairwise comparisons among ChatGPT, Gemini, DeepSeek, and Grok across the five evaluation dimensions.

Panel A. Global Quality Score

Comparison	Mean A	Mean B	Median (A-B)	n_eff	W	Z	Raw p	Adjusted p	|r|	r_rb (95% CI)	Significant	Direction
ChatGPT - Gemini	3.899	4.071	0.000	20	50.5	-2.061	0.039	0.236	0.461	-0.519 (-0.948 to -0.040)	No	Gemini higher
ChatGPT - DeepSeek	3.899	4.000	-0.333	29	160.5	-1.246	0.213	1.000	0.231	-0.262 (-0.658 to 0.138)	No	DeepSeek higher
ChatGPT - Grok	3.899	3.707	0.000	25	73.0	-2.425	0.015	0.092	0.485	0.551 (0.140 to 0.842)	No	ChatGPT higher
Gemini - DeepSeek	4.071	4.000	0.000	18	71.0	-0.640	0.522	1.000	0.151	0.170 (-0.382 to 0.652)	No	Gemini higher
Gemini - Grok	4.071	3.707	0.333	24	16.5	-3.918	<0.001	<0.001	0.800	0.890 (0.649 to 1.000)	Yes	Gemini higher
DeepSeek - Grok	4.000	3.707	0.333	26	45.5	-3.331	<0.001	0.005	0.653	0.741 (0.396 to 0.978)	Yes	DeepSeek higher

Panel B. Clinical accuracy

Comparison	Mean A	Mean B	Median (A-B)	n_eff	W	Z	Raw p	Adjusted p	|r|	r_rb (95% CI)	Significant	Direction
ChatGPT - Gemini	4.000	4.192	-0.333	22	54.5	-2.352	0.019	0.112	0.501	-0.569 (-0.965 to -0.137)	No	Gemini higher
ChatGPT - DeepSeek	4.000	4.101	-0.333	24	92.5	-1.655	0.098	0.587	0.338	-0.383 (-0.792 to 0.053)	No	DeepSeek higher
ChatGPT - Grok	4.000	3.899	0.000	26	141.0	-0.882	0.378	1.000	0.173	0.197 (-0.254 to 0.581)	No	ChatGPT higher
Gemini - DeepSeek	4.192	4.101	0.000	14	45.5	-0.446	0.655	1.000	0.119	0.133 (-0.464 to 0.705)	No	Gemini higher
Gemini - Grok	4.192	3.899	0.333	24	18.0	-3.826	<0.001	<0.001	0.781	0.880 (0.680 to 1.000)	Yes	Gemini higher
DeepSeek - Grok	4.101	3.899	0.333	22	33.0	-3.074	0.002	0.013	0.655	0.739 (0.363 to 1.000)	Yes	DeepSeek higher

Panel C. Ateşman readability

Comparison	Mean A	Mean B	Median (A-B)	n_eff	W	Z	Raw p	Adjusted p	|r|	r_rb (95% CI)	Significant	Direction
ChatGPT - Gemini	54.647	46.984	8.274	33	101.0	-3.207	0.001	0.008	0.558	0.640 (0.305 to 0.914)	Yes	ChatGPT higher
ChatGPT - DeepSeek	54.647	56.215	1.274	33	269.0	-0.205	0.837	1.000	0.036	-0.041 (-0.412 to 0.358)	No	DeepSeek higher
ChatGPT - Grok	54.647	56.638	0.533	33	256.0	-0.438	0.662	1.000	0.076	-0.087 (-0.472 to 0.316)	No	Grok higher
Gemini - DeepSeek	46.984	56.215	-10.825	33	58.0	-3.976	<0.001	<0.001	0.692	-0.793 (-1.000 to -0.537)	Yes	DeepSeek higher
Gemini - Grok	46.984	56.638	-8.879	33	15.0	-4.744	<0.001	<0.001	0.826	-0.947 (-1.000 to -0.832)	Yes	Grok higher
DeepSeek - Grok	56.215	56.638	0.159	33	275.0	-0.098	0.922	1.000	0.017	0.020 (-0.365 to 0.415)	No	Grok higher

Panel D. PEMAT understandability

Comparison	Mean A	Mean B	Median (A-B)	n_eff	W	Z	Raw p	Adjusted p	|r|	r_rb (95% CI)	Significant	Direction
ChatGPT - Gemini	78.425	78.790	0.000	16	57.5	-0.547	0.585	1.000	0.137	-0.154 (-0.744 to 0.433)	No	Gemini higher
ChatGPT - DeepSeek	78.425	76.068	0.000	12	25.0	-1.114	0.265	1.000	0.322	0.359 (-0.303 to 0.944)	No	ChatGPT higher
ChatGPT - Grok	78.425	75.537	0.000	16	14.0	-2.821	0.005	0.029	0.705	0.794 (0.364 to 1.000)	Yes	ChatGPT higher
Gemini - DeepSeek	78.790	76.068	0.000	18	58.0	-1.215	0.224	1.000	0.286	0.322 (-0.231 to 0.832)	No	Gemini higher

Comparison	Mean A	Mean B	Median (A-B)	n_eff	W	Z	Raw p	Adjusted p	|r|	r_rb (95% CI)	Significant	Direction
Gemini - Grok	78.790	75.537	2.564	23	22.5	-3.549	<0.001	0.002	0.740	0.837 (0.547 to 1.000)	Yes	Gemini higher
DeepSeek - Grok	76.068	75.537	0.000	14	26.5	-1.638	0.101	0.608	0.438	0.495 (-0.077 to 1.000)	No	DeepSeek higher

Panel E. PEMAT actionability

Comparison	Mean A	Mean B	Median (A-B)	n_eff	W	Z	Raw p	Adjusted p	|r|	r_rb (95% CI)	Significant	Direction
ChatGPT - Gemini	77.576	79.259	0.000	8	6.0	-1.691	0.091	0.545	0.598	-0.667 (-1.000 to 0.067)	No	Gemini higher
ChatGPT - DeepSeek	77.576	76.566	0.000	9	16.5	-0.715	0.475	1.000	0.238	-0.267 (-1.000 to 0.500)	No	ChatGPT higher
ChatGPT - Grok	77.576	73.939	0.000	15	28.5	-1.801	0.072	0.430	0.465	0.525 (0.000 to 0.962)	No	ChatGPT higher
Gemini - DeepSeek	79.259	76.566	0.000	8	12.5	-0.775	0.438	1.000	0.274	0.306 (-0.536 to 1.000)	No	Gemini higher
Gemini - Grok	79.259	73.939	0.000	12	0.0	-3.083	0.002	0.012	0.890	1.000 (1.000 to 1.000)	Yes	Gemini higher
DeepSeek - Grok	76.566	73.939	0.000	14	26.5	-1.655	0.098	0.588	0.442	0.495 (-0.086 to 1.000)	No	DeepSeek higher

Notes: All comparisons used two-sided Wilcoxon signed-rank tests based on N = 33 matched dental questions. Bonferroni-adjusted p values account for the six pairwise comparisons within each outcome. n_eff denotes the number of non-zero paired differences. W is the Wilcoxon signed-rank statistic. The standardized effect size was calculated as |r| = |Z|/sqrt(n_eff). The matched-pairs rank-biserial correlation was calculated as r_rb = (W_plus - W_minus)/(W_plus + W_minus); positive values favor the first-listed model and negative values favor the second-listed model. Confidence intervals for r_rb were estimated using 5,000 paired question-level bootstrap samples. The Ateşman results are based on the final deterministic response-level recalculation. PEMAT-U, Patient Education Materials Assessment Tool-Understandability; PEMAT-A, Patient Education Materials Assessment Tool-Actionability.

Supplementary Table S4

Exploratory multidimensional Pareto analysis across clinical accuracy, global quality, readability, understandability, and actionability.

Panel A. Model-level performance profiles and probability of remaining non-dominated

Model	GQS	Clinical accuracy	Ateşman	PEMAT-U	PEMAT-A	Dominated at observed means	Bootstrap non-dominated probability
ChatGPT	3.899	4.000	54.647	78.425	77.576	No	90.44%
Gemini	4.071	4.192	46.984	78.790	79.259	No	99.01%
DeepSeek	4.000	4.101	56.215	76.068	76.566	No	96.36%
Grok	3.707	3.899	56.638	75.537	73.939	No	60.71%

Panel B. Ordered pairwise dominance probabilities

Potentially dominating model	Potentially dominated model	Dominance at observed means	Bootstrap dominance probability
ChatGPT	Gemini	No	0.03%
ChatGPT	DeepSeek	No	3.51%
ChatGPT	Grok	No	16.18%
Gemini	ChatGPT	No	0.13%
Gemini	DeepSeek	No	0.00%
Gemini	Grok	No	0.00%
DeepSeek	ChatGPT	No	9.47%
DeepSeek	Gemini	No	0.96%
DeepSeek	Grok	No	30.73%
Grok	ChatGPT	No	0.01%
Grok	Gemini	No	0.00%
Grok	DeepSeek	No	0.19%

Notes: All five dimensions were oriented so that higher values indicated better performance. Model A was defined as dominating Model B when A performed at least as well as B in all five dimensions and strictly better in at least one dimension. At the observed mean level, no model dominated another; therefore, all four models remained in the non-dominated set. Bootstrap probabilities were estimated using 10,000 paired question-level resamples. The PEMAT-U and PEMAT-A components were recalculated using standard applicability-based PEMAT-P scoring with N/A items excluded from response-specific denominators. This post hoc analysis was exploratory and hypothesis-generating.
