# Supplementary material for: Quality, Readability, Understandability, and Actionability of AI Chatbots’ Responses to Dental Patient Questions: A Comparative Evaluation
Source: Diagnostics (Basel). 2026 Oct 21;16(20):3430. doi: 10.3390/diagnostics16203430 (PMC13703409; doi:10.3390/diagnostics16203430)
Supplement: Supplementary file 1 [file diagnostics-16-03430-s001.zip › Supplementary Material Table S1.pdf]

Supplementary Material

Supplementary Table S1. Verbatim dental patient questions submitted to each AI chatbot model.

Note. The Turkish question text represents the verbatim standalone prompt submitted to ChatGPT 5.2, Gemini, DeepSeek V3, and Grok 3 in a new chat session. English translations are provided for reader convenience. No follow-up prompts, corrective prompts, regeneration requests, or additional instructions regarding response length, tone, reading level, citation use, or formatting were used.

Question No.	Clinical category	Verbatim prompt submitted to each model (Turkish; English translation)
1	Tooth Pain and Infections	Diş ağrım var ama çürük görünmüyor, neden olabilir?<br>English translation: I have a toothache, but no cavity is visible. What could be the reason?
2	Tooth Pain and Infections	Kanal tedavisinden sonra ağrı normal mi?<br>English translation: Is pain after root canal treatment normal?
3	Tooth Pain and Infections	Apse oluştu, kendiliğinden geçer mi?<br>English translation: An abscess has formed. Can it go away on its own?
4	Tooth Pain and Infections	Antibiyotiksiz apse geçer mi?<br>English translation: Can an abscess heal without antibiotics?
5	Tooth Pain and Infections	Gece dişim ağrıyor, neden?<br>English translation: My tooth hurts at night. Why?
6	Periodontal and Gingival Problems	Diş etlerim kanıyor, ne yapmalıyım?<br>English translation: My gums are bleeding. What should I do?
7	Periodontal and Gingival Problems	Diş eti çekilmesi geri döner mi?<br>English translation: Can gum recession reverse?
8	Periodontal and Gingival Problems	Diş eti şişliği neden olur?<br>English translation: What causes gum swelling?
9	Periodontal and Gingival Problems	Ağız kokusuna diş eti hastalığı neden olur mu?<br>English translation: Can gum disease cause bad breath?
10	Tooth Extraction and Post-Extraction Care	Diş çekiminden sonra ağrı kaç gün sürer?<br>English translation: How many days does pain last after tooth extraction?
11	Tooth Extraction and Post-Extraction Care	Kuru soket nedir, nasıl anlaşılır?<br>English translation: What is dry socket, and how can it be recognized?
12	Tooth Extraction and Post-Extraction Care	Çekim sonrası ne zaman yemek yiyebilirim?<br>English translation: When can I eat after tooth extraction?
13	Tooth Extraction and Post-Extraction Care	Sigara çekim sonrası kaç gün içilmez?<br>English translation: For how many days should smoking be avoided after tooth extraction?
14	Impacted Teeth and Third Molars	20 yaş dişim gömülü, çektirmem gerekir mi?<br>English translation: My wisdom tooth is impacted. Does it need to be extracted?
15	Impacted Teeth and Third Molars	Gömülü diş ameliyatı zor mu?<br>English translation: Is impacted tooth surgery difficult?
16	Impacted Teeth and Third Molars	20’lik diş çekimi sonrası yüz şişer mi?

Question No.	Clinical category	Verbatim prompt submitted to each model (Turkish; English translation)
		English translation: Does the face swell after wisdom tooth extraction?
17	Impacted Teeth and Third Molars	20'lik dişim ağrımıyor, çektirmesem olur mu?<br>English translation: My wisdom tooth does not hurt. Is it okay if I do not have it extracted?
18	Dental Implants, Bridges, and Prostheses	İmplant ağrılı bir işlem mi?<br>English translation: Is dental implant treatment a painful procedure?
19	Dental Implants, Bridges, and Prostheses	İmplant ömür boyu kalır mı?<br>English translation: Do dental implants last for a lifetime?
20	Dental Implants, Bridges, and Prostheses	Köprü mü, implant mı daha iyi?<br>English translation: Which is better, a bridge or an implant?
21	Dental Implants, Bridges, and Prostheses	Protezler neden oynar veya yara yapar?<br>English translation: Why do dentures move or cause sores?
22	Dental Esthetics and Tooth Whitening	Diş beyazlatma zararlı mı?<br>English translation: Is tooth whitening harmful?
23	Dental Esthetics and Tooth Whitening	Lamine (yaprak porselen) kalıcı mı?<br>English translation: Are laminate veneers permanent?
24	Dental Esthetics and Tooth Whitening	Diş taşlarını temizletmek dişleri aşındırır mı?<br>English translation: Does dental calculus removal wear down the teeth?
25	Dental Esthetics and Tooth Whitening	Dişlerimi evde nasıl beyazlatabilirim?<br>English translation: How can I whiten my teeth at home?
26	Pediatric Dental Problems	Süt dişi erken çekilirse ne olur?<br>English translation: What happens if a primary tooth is extracted early?
27	Pediatric Dental Problems	Çocuğumun dişi sallanıyor ama düşmüyor, çekilmeli mi?<br>English translation: My child's tooth is loose but does not fall out. Should it be extracted?
28	Pediatric Dental Problems	Flor uygulaması zararlı mı?<br>English translation: Is fluoride application harmful?
29	Pediatric Dental Problems	Diş fırçalama alışkanlığını nasıl kazandırırım?<br>English translation: How can I help my child develop a toothbrushing habit?
30	General Information and Preventive Dentistry	Ağız kokusunu nasıl önleyebilirim?<br>English translation: How can I prevent bad breath?
31	General Information and Preventive Dentistry	Günde kaç kez diş fırçalamalıyım?<br>English translation: How many times a day should I brush my teeth?
32	General Information and Preventive Dentistry	Diş ipi gerçekten gerekli mi?<br>English translation: Is dental floss really necessary?
33	General Information and Preventive Dentistry	Ağız gargarası diş fırçalamanın yerini alır mı?<br>English translation: Can mouthwash replace toothbrushing?
